# PDE5 Inhibition Suppresses Ventricular Arrhythmias by Reducing SR Ca^2+^ Content

**DOI:** 10.1161/CIRCRESAHA.121.318473

**Published:** 2021-07-12

**Authors:** David C. Hutchings, Charles M. Pearman, George W.P. Madders, Lori S. Woods, David A. Eisner, Katharine M. Dibb, Andrew W. Trafford

**Affiliations:** Unit of Cardiac Physiology, Division of Cardiovascular Sciences, Faculty of Biology Medicine and Health, University of Manchester, Manchester Academic Health Sciences Centre, United Kingdom.

**Keywords:** calcium, long QT syndrome, sildenafil citrate

## Abstract

PDE5 (phosphodiesterase 5) inhibition reduces the occurrence of ventricular arrhythmias following myocardial ischemia. However, the mechanisms of the antiarrhythmic effects of PDE5 inhibition are unknown. Diastolic calcium (Ca^2+^) waves lead to arrhythmias by inducing delayed afterdepolarizations (DADs). Ca^2+^ waves are initiated when sarcoplasmic reticulum (SR) Ca^2+^ content reaches a threshold level and the SR releases Ca^2+^ spontaneously and generates a depolarizing inward sodium-calcium exchange current.

**Objective:**

To determine the effects of PDE5 inhibition on the propensity for ventricular arrhythmias in a proarrhythmic large animal model and establish the role of alterations of intracellular Ca^2+^ cycling/SR Ca^2+^ content.

**Methods and Results:**

Arrhythmia burden, monophasic action potentials, and beat-to-beat variability of repolarization were measured in a sheep model using the *I*_Kr_ inhibitor dofetilide to induce QT prolongation and arrhythmia. Ca^2+^ transients, Ca^2+^ waves, and SR Ca^2+^ content were measured in isolated ventricular myocytes. PDE5 inhibition was achieved using acute application of sildenafil, and PKG (protein kinase G) was inhibited with KT5823. PDE5 inhibition reduced beat-to-beat variability of repolarization and suppressed afterdepolarizations, premature ventricular complexes, and torsade de pointes in vivo. In single cells, dofetilide-induced delayed afterdepolarizations and triggered action potentials were suppressed by PDE5 inhibition. PDE5 inhibition decreased Ca^2+^ wave frequency in all cells and abolished waves in 12 of 22 cells. A decrease in SR Ca^2+^ uptake, increased trans-sarcolemmal Ca^2+^ efflux, and reduced trans-sarcolemmal Ca^2+^ influx led to a reduction of SR Ca^2+^ content and Ca^2+^ wave abolition. These effects were dependent on PKG activation.

**Conclusions:**

PDE5 inhibition acutely suppresses triggered ventricular arrhythmias in vivo, and cellular data suggests this occurs via suppression of cellular Ca^2+^ waves. These novel antiarrhythmic properties of PDE5 inhibition are mediated by a reduction of SR Ca^2+^ content and are PKG dependent.

## Abstract 2

Supplemental Digital Content is available in the text.

**Meet the First Author, see p 601**

**Editorial, see p 666**

Prolongation of action potential (AP) duration (APD) and thence QT interval are established causes of ventricular arrhythmias.^1,2^ Perturbations to cellular Ca^2+^ cycling may contribute to arrhythmias in long QT syndromes by 2 mechanisms: (i) reactivation of L-type Ca^2+^ channel and (ii) spontaneous Ca^2+^ release from the sarcoplasmic reticulum (SR).^3–6^ During the AP, Ca^2+^ is normally released from the SR in response to trans-sarcolemmal Ca^2+^ entry on the L-type Ca^2+^ channel (L-type Ca^2+^ current [*I*_Ca-L_]).^7,8^ However, under conditions of SR Ca^2+^ overload, Ca^2+^ release from the SR can occur independently from the AP thereby generating propagating Ca^2+^ waves.^9,10^ During these Ca^2+^ waves, some Ca^2+^ is pumped out of the cell by the electrogenic NCX (Na^+^-Ca^2+^ exchanger), resulting in membrane depolarization and triggered activity/delayed afterdepolarizations (DADs).^11–13^ These data support the concept of a threshold SR Ca^2+^ content^14–16^ as a key driver of Ca^2+^-dependent arrhythmias including both early and delayed afterdepolarizations in diverse conditions including digitalis toxicity,^11,17^ catecholaminergic polymorphic ventricular tachycardia,^18,19^ heart failure,^20^ and long QT syndromes.^21^

β-AR (β-adrenergic receptor) stimulation is known to exacerbate arrhythmias, and activation of PKG (protein kinase G) is widely accepted to antagonize the effects of β-AR stimulation.^22–24^ Therefore, the initial aim of this study was to determine whether activation of the PKG pathway, using the PDE5 (phosphodiesterase 5) inhibitor sildenafil to increase cGMP, was protective against long QT arrhythmias and whether this involved alterations in Ca^2+^ release from the SR and thence prevention of triggered arrhythmias.

Using an integrative whole animal and cellular approach, we have investigated the impact of PDE5 inhibition in a drug-induced sheep arrhythmia model. In particular, we sought to (1) establish the acute effects of sildenafil on ventricular arrhythmias, (2) determine the cellular correlates for its antiarrhythmic effects and, (3) understand the underlying mechanisms of PDE5 inhibition through analysis of cellular Ca^2+^ cycling. We found that sildenafil dramatically reduced the incidence and delayed the occurrence of afterdepolarizations, premature ventricular complexes (PVCs), and Torsades de Pointes (TdP) in vivo, and these effects were attributable to a PKG-dependent effect on Ca^2+^ waves and involved a reduction in SR Ca^2+^ content.

## Methods

### Data Availability

The data that support the findings of this study are available from D.C.H. upon reasonable request. An expanded Methods section is available in the Data Supplement.

All procedures involving the use of animals were performed in accordance with The United Kingdom Animals (Scientific Procedures) Act, 1986 and European Union Directive 2010/63. Institutional approval was obtained from The University of Manchester Animal Welfare and Ethical Review Board. The reporting of animals in experimental studies accords with the ARRIVE (Animal Research: Reporting of In Vivo Experiments) guidelines.^25^

### In Vivo Studies

A model of dofetilide-induced QT prolongation was used in 11 female Welsh mountain sheep (≈18 months of age; 33.2±1.1 kg). All procedures were performed under general anesthesia (2%–3% isoflurane in oxygen) and preoperative analgesia (meloxicam, 0.5 mg/kg, s.c.) and antibiosis (enrofloxacin, 2.5 mg/kg, subcutaneously.) provided. An endocardial DF-1 defibrillation lead with superior vena cava coil was positioned at the right ventricular apex and connected to a Medtronic implantable defibrillator to allow cardioversion of sustained arrhythmias. Arterial blood pressure (BP) was recorded every 2 to 3 minutes via forelimb plethysmography. Under continuous ECG monitoring, ventricular arrhythmias were induced by intravenous administration of the *I*_Kr_ blocker dofetilide (Stratech Scientific, Ltd, United Kingdom) in increments of 3 µg/kg until episodes of nonsustained ventricular tachycardia/TdP occurred (maximum total dose of 15 µg/kg). Animals were randomized (using a coin toss) to receive either 10 mg sildenafil bolus (Pfizer, United States) or saline control of the same volume (12.5 mL) and the administering investigator blinded as to which was given. Arrhythmia analysis was performed according to a predefined protocol. In the event of sustained ventricular tachycardia/TdP (duration, >20 s) or ventricular fibrillation, sinus rhythm was restored by intracardiac defibrillation (35 J). In 6 animals, left ventricular endocardial monophasic APs were recorded using an ablation catheter (Boston Scientific, MA, United States) advanced to the left ventricular apex under fluoroscopic guidance. No animals were excluded from the analysis.

### Cellular Studies

Single left ventricular myocytes were isolated from 45 female adult Welsh mountain sheep (≈18 months of age) using a collagenase and protease digestion technique described previously.^26,27^ The perforated patch technique was used to measure APs under current clamp control, and dofetilide (5 µmol/L) and low K^+^ (2 mmol/L) were used to induce afterdepolarizations and ectopic APs. *I*_Ca-L_ and NCX current were measured using the whole-cell voltage clamp technique as reported previously.^27^ Intracellular Ca^2+^ concentration ([Ca^2+^]_i_) was measured using Fura-2 (K_5_ salt; 100 µmol/L).^28^ Ca^2+^ waves were induced in single ventricular myocytes by increasing the external Ca^2+^ concentration (10–15 mmol/L).^15^ SR Ca^2+^ content was quantified by application of 10 mmol/L caffeine and integration of the resulting NCX current as described previously.^29^ Cells did not tolerate multiple caffeine applications in the presence of this high Ca^2+^ concentration; therefore, the majority of comparisons of SR content were made between different cells (ie, unpaired). PDE5 inhibition was achieved using sildenafil (1 µmol/L; Sigma, United Kingdom). In some experiments, a lower concentration of sildenafil (20 nmol/L) was also tested, where indicated in the text (Figure 4D through 4F; Figure IX in the Data Supplement). PKG was inhibited by preincubation for at least 30 minutes using KT5823 (1 µmol/L; Abcam, United Kingdom). SERCA (sarcoplasmic endoplasmic reticulum Ca^2+^ ATPase) was inhibited with thapsigargin (5 µmol/L; Sigma, United Kingdom). Sildenafil, thapsigargin, dofetilide, and KT5823 were prepared in DMSO (dimethylsulfoxide; final concentration not exceeding 0.1% v/v). In all experiments, a vehicle control was used with the same concentration of DMSO. For all research materials, please see the Major Resources Table in the Data Supplement. All cellular experiments were performed at 37 °C.

**Figure 1.**
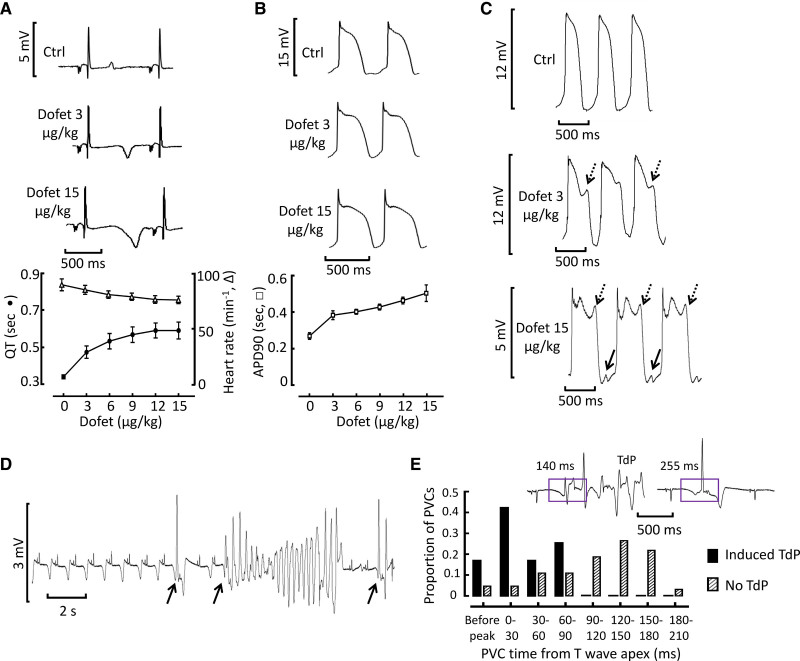
**/Kr block with dofetilide prolongs ventricular repolarization leading to afterdepolarizations and triggered ventricular arrhythmias.A**, Effect of dofetilide (Dofet) on QT interval and heart rate. Representative paired surface ECG recordings (**top**) and mean data (**bottom**). Effect of dofetilide on QT interval is significant at all concentrations (3 µg/kg, P=0.02; 6 µg/kg, P=0.02; 9 µg/kg, *P*=0.01; 12 µg/kg, *P*=0.006; 15 µg/kg, *P*=0.01; CTRL, control). Effect of dofetilide on HR (heart rate) is significant at ≥12 µg/kg (12 µg/kg, *P*=0.04; 15 µg/kg, *P*=0.03). For QT interval and HR, paired data from n=6 animals, 1-way ANOVA. **B**, Effect of dofetilide on action potential duration. **Top**, Representative paired monophasic action potential (MAP) recordings. **Bottom**, Mean data. Effect is significant at concentrations ≥9 µg/kg (3 µg/kg, *P*=0.18; 6 µg/kg, *P*=0.18; 9 µg/kg, *P*=0.018; 12–15 µg/kg, P<0.001; paired data from n=5 animals; Friedman test). **C**, MAP traces recorded in sinus rhythm showing dofetilide-induced early afterdepolarizations and delayed afterdepolarizations (indicated by dashed and continuous arrows, respectively). **D**, Representative surface ECG recording showing Torsades de Pointes (TdP) triggered by premature ventricular complex (PVC) arising on the preceding T wave (see arrows). **E**, Summary data showing PVC timing in relation to T-wave apex. PVCs inducing TdP occur earlier and are more closely aligned with the apex of the T wave compared with PVCs that do not induce TdP. Upper inset shows representative PVCs and their timing from the start of preceding T wave, with PVC causing TdP (**left**) and PVC which does not cause TdP (**right**). APD indicates action potential duration.

**Figure 2.**
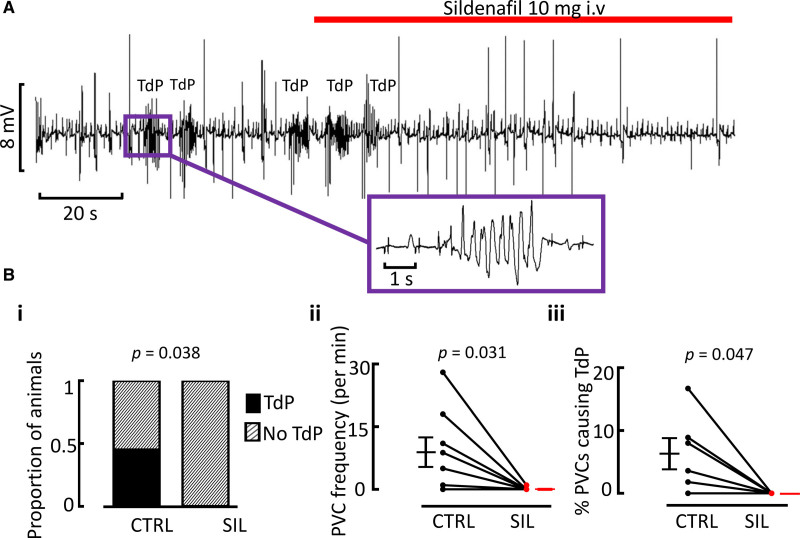
**Sildenafil suppresses dofetilide-induced ventricular arrhythmias.A**, Representative surface ECG recording from a sheep displaying dofetilide-induced premature ventricular complexes (PVCs) and Torsades de Pointes (TdP). A 10 milligram bolus intravenous injection of sildenafil given as shown. **B**, Mean data showing effect of sildenafil on the (**Bi**) incidence of TdP, (**Bii**) frequency of PVCs (at 10 min after sildenafil), and (**Biii**) probability of PVCs causing TdP. **Bi**, For Fisher's exact test from 11 animals (control) and 9 animals (sildenafil). **Bii**, For Wilcoxon signed-rank test on paired data from 8 animals. **Biii**, For paired *t* test from 6 animals.

**Figure 3.**
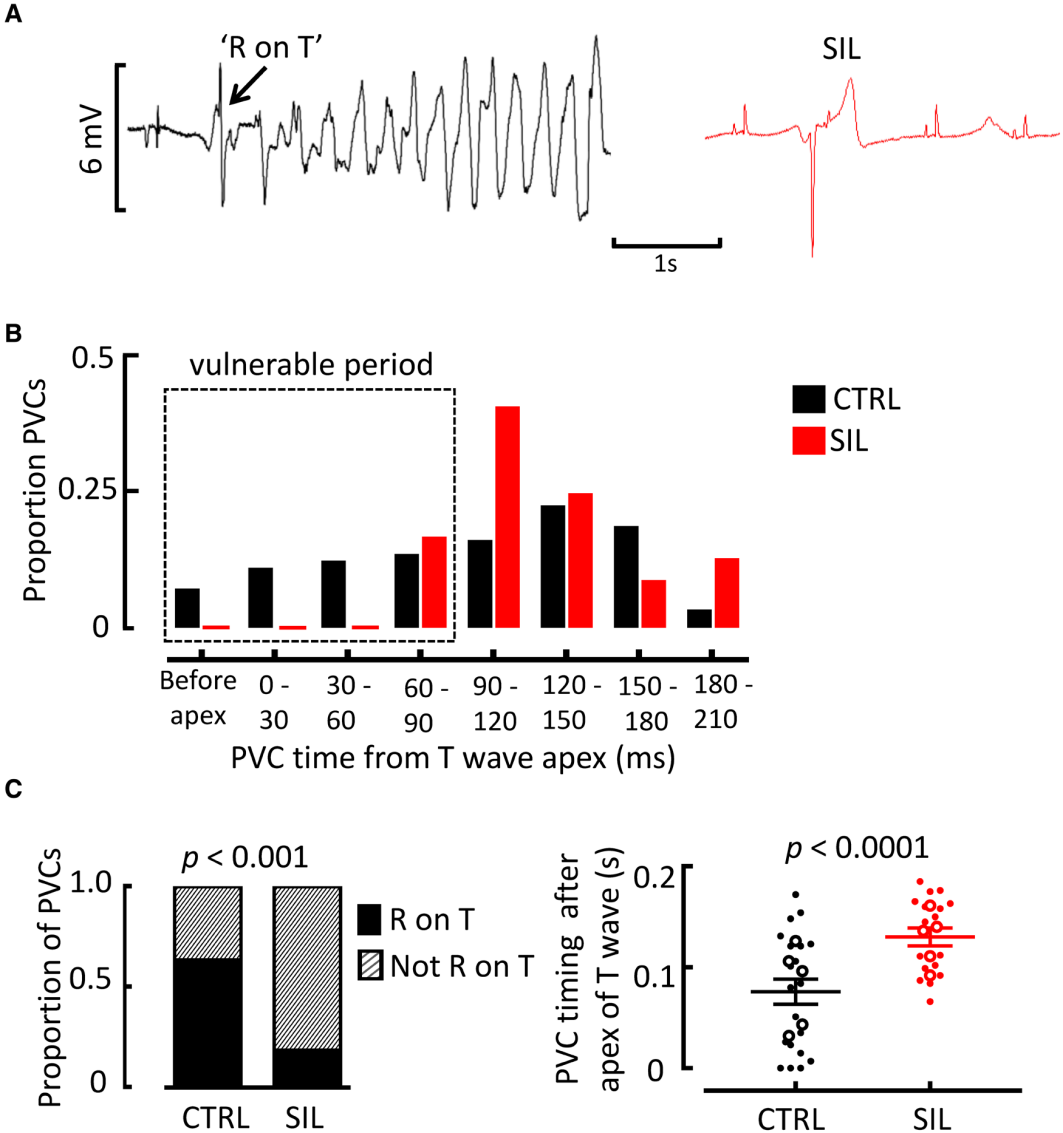
**Sildenafil delays premature ventricular complexes (PVCs) relative to T-wave apex.A**, Representative surface ECG recordings of a PVC in control with R on T effect (**left**) and in the presence of sildenafil (**right**). **B**, Effect of sildenafil (10 mg IV bolus) on PVC timing in relation to the preceding T wave. Sildenafil delays PVCs such that they occur further from the T-wave apex and beyond the vulnerable period. **C**, **Left**, Mean data on proportion of PVCs occurring on T wave in sildenafil (control, n=84 PVCs from 8 animals; sildenafil, n=33 PVCs from 5 animals; χ^2^ test). **C**, **Right**, Mean data summarizing effect of sildenafil on timing of PVCs after the apex of the preceding T wave. Open circles indicate mean data for each animal, and closed circles indicate individual PVCs. Linear mixed modeling *t* test from n=5 animals.

### Statistics

Data are presented as mean±SEM for n cells for the myocyte experiments and N animals for in vivo studies. All analysis was performed using GraphPad Prism, version 7.00. Normality of data distribution (raw or following logarithmic transformation) was tested with a D’Agostino-Pearson or Shapiro-Wilk test. For normally distributed data, differences between treatment groups were determined using paired or unpaired *t* tests as indicated in the figure legends. In some figures where data were ratioed to control values, significance was assessed with a 1-sample *t* test. Categorical variables were compared between groups using the Fisher exact or χ^2^ tests as appropriate (Figures 2B, 3C, 5A, and 8C). Nonparametric tests were used when data were not normally distributed (see figure legends). Where sample size was too small to determine normality (<6), a nonparametric test was used. No correction has been made for multiple testing. Exact *P* values are presented at *P*>0.001, and differences were considered statistically significant at *P*<0.05.

## Results

### Mechanism of Triggered Arrhythmia in a Dofetilide Model of QT Prolongation

Administration of the *I*_Kr_ blocker dofetilide resulted in dose-dependent prolongation of the QT interval (Figure 1A),^30^ an increase in T wave duration and altered T-wave morphology, and small reductions in heart rate. Systolic arterial pressure was reduced by dofetilide, whereas diastolic pressure was unchanged (Table I in the Data Supplement). Accompanying the effect on the QT interval was a dose-dependent prolongation of the endocardial monophasic APD (Figure 1B), associated with the occurrence of afterdepolarizations (Figure 1C) in 5 of the 6 animals where monophasic APs were recorded (*P*=0.028; Figure 1C). In all these 5 animals, afterdepolarizations preceded the onset of arrhythmias on the surface ECG.

PVCs developed in 8 of 11 animals, with TdP occurring in 5 animals (Figure 1D). Each episode of TdP was preceded by a PVC falling on the preceding T wave (R on T phenomenon). However, 56.3% of PVCs falling on a T wave did not cause TdP. The importance of PVC timing in relation to the preceding T wave and the occurrence of TdP is depicted graphically in Figure 1E. Compared with non-TdP causing PVCs, those causing TdP arose earlier and were more tightly clustered in relation to the T-wave apex (Figure 1E; 33.7±9.7 ms after T apex versus 111.5±5.6 ms; *P*<0.0001). Thus, in agreement with previous work, the interval between the T wave and subsequent PVC plays a key role in determining likelihood of triggering TdP.^31–34^

### PDE5 Inhibitor Sildenafil Suppresses Ventricular Arrhythmias In Vivo

In all the 5 animals showing TdP, intravenous sildenafil abolished dofetilide-induced TdP within 90 s of administration (Figure 2A and 2Bi; *P*=0.008). In all 8 animals where dofetilide induced PVCs, sildenafil reduced PVC frequency (by 39.4±13.4%; *P*=0.043). The antiarrhythmic effect of sildenafil persisted for the duration of the experiment (up to 15 minutes) with PVC frequency decreasing by 72.5±18.3% between 120 and 180 s (*P*=0.017) and by 90.2±6.5% at 5 minutes (Figure 2Bii; *P*<0.0005). Saline alone, as a vehicle control for sildenafil, had no effect on PVC frequency or TdP incidence. Sildenafil also reduced the beat-to-beat variability in QT interval (Figure I in the Data Supplement). Other ECG parameters were unaffected by sildenafil (Table II in the Data Supplement). Sildenafil suppression of TdP appeared to have 2 key components: (1) a reduced frequency of PVCs (Figure 2Bii) and (2) a decrease in the probability that a PVC causes TdP (Figure 2Biii).

**Figure 4.**
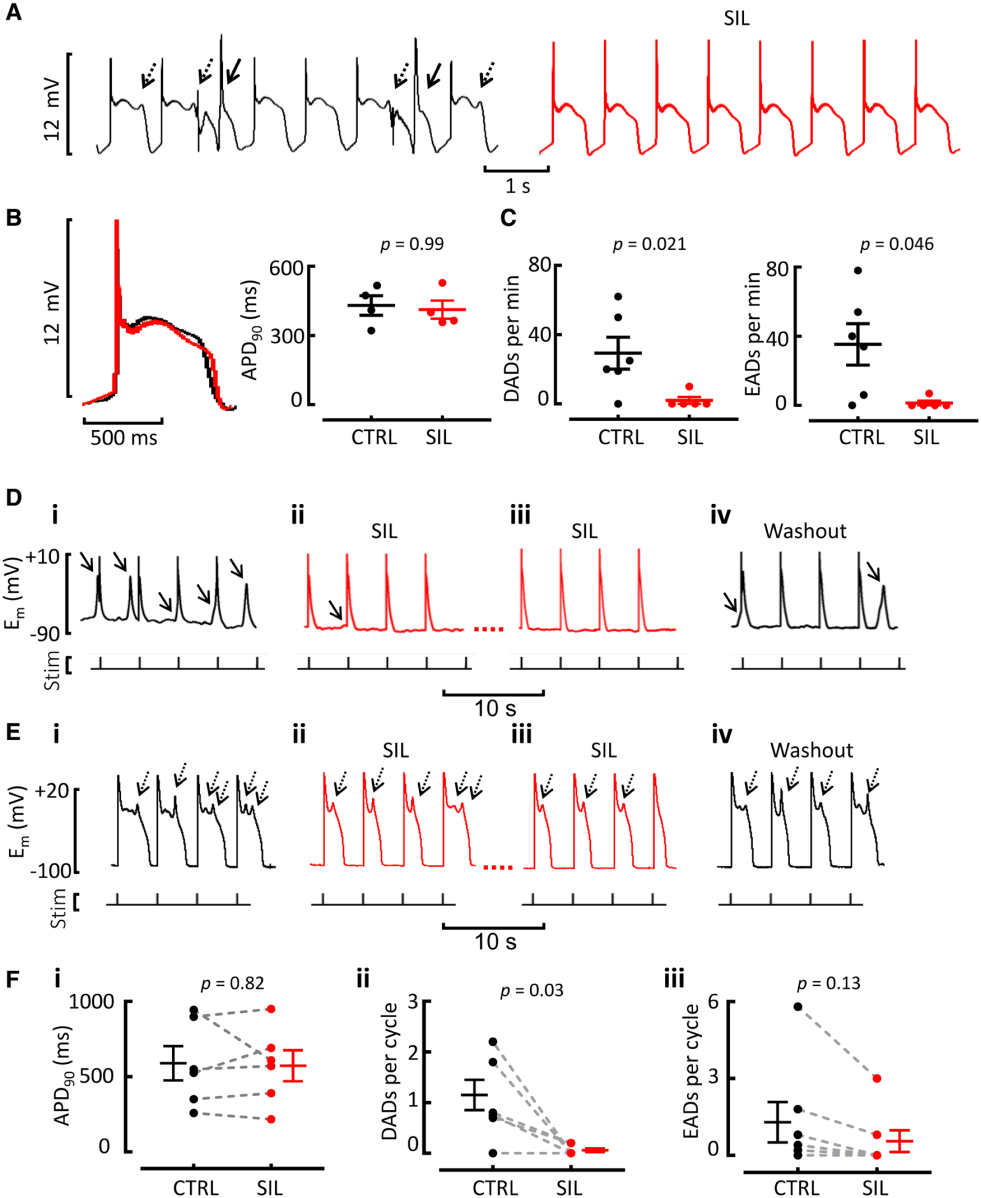
**Sildenafil suppresses afterdepolarizations in vivo and isolated myocytes without modifying monophasic action potential duration.A**, Representative paired in vivo monophasic action potential (MAP) recordings in sinus rhythm in control (**left**) and sildenafil (**right**). Early afterdepolarizations (EADs) and delayed afterdepolarizations (DADs) are indicated by dashed and continuous arrows, respectively. **B**, Effect of sildenafil on monophasic action potential duration. Representative MAP traces from the same animal (**left**) and summary data (**right**). Paired data on APD^90^ from 4 animals, Wilcoxon signed-rank test. **C**, Effect of sildenafil on afterdepolarizations. Mean data for DADs (**left**) and EADs (**right**). Unpaired data on afterdepolarizations from 5 to 6 animals, Mann-Whitney *U* test. **Di**, Example paired current clamp (0.25 Hz stimulation) recordings of a myocyte showing DADs when exposed to dofetilide and low K^+^. DADs are indicated by arrows. **Dii**, After a 10 s exposure to sildenafil (20 nmol/L). **Diii**, After a 40 s exposure to sildenafil. **Div**, Washout of sildenafil. **Ei**, Example paired current clamp recordings of a myocyte displaying EADs in dofetilide and low K^+^. Dashed arrows indicate EADs. **Eii**, Following a 10 s exposure to sildenafil. **Eiii**, Following a 40 s exposure to sildenafil. **Div**, Following washout of sildenafil. **F**, Summary data for cells in dofetilide and low K^+^. Action potential duration, recorded from cells not showing EADs (**Fi**), DADs (**Fii**), and EADs (**Fiii**). For APD^90^, paired data from 6 cells/3 animals, *t* test. For DADs and EADs, paired data from 7 cells/3 animals, Wilcoxon signed-rank test. APD indicates action potential duration.

To understand why PVCs in sildenafil are less likely to induce TdP, we next investigated the effect of sildenafil on PVC timing in relation to the T wave (Figure 3). Sildenafil delayed the timing of PVCs such that the fraction occurring in the vulnerable window around the apex of the T wave was reduced. The reduction of R on T behavior was not due to any sildenafil-induced changes in QT duration (Figure II in the Data Supplement). Additionally, sildenafil had no effect on left ventricular endocardial monophasic APD (Figure 4A and 4B). These experiments did, however, demonstrate the importance of sildenafil reducing the occurrence of DADs and early afterdepolarizations (EADs; Figure 4C). Because sildenafil decreased heart rate, we investigated the effect of sildenafil on ECG parameters and monophasic APD in 3 atrially paced animals (RR interval, 500 ms). Again, sildenafil had no statistically significant effect on QT interval or monophasic APD (QT: control, 416±12 ms; sildenafil, 415±17 ms; APD_90_: control, 341±18 ms; sildenafil, 310±9 ms).

To determine whether the antiarrhythmic action of sildenafil was related to hemodynamic effects, BP was recorded throughout the study period (every 2–3 minutes). While sildenafil reduced arterial BP (Figure III in the Data Supplement; *P*=0.02), the hypotensive effects of sildenafil were maximal at a dose of 2.5 mg—a level at which there was no significant effect on DAD or EAD frequency. Furthermore, additional doses of sildenafil decreased afterdepolarization frequency but had no additive effect on BP. Thus, we conclude that sildenafil (1) acts independently of the QT interval and BP in suppressing arrhythmias, (2) suppresses afterdepolarizations, reducing PVC frequency, and (3) reduces R on T through the later timing of PVCs.

### Cellular Mechanisms Responsible for Suppression of Dofetilide-Induced Arrhythmias

We next sought to establish whether the antiarrhythmic effects of sildenafil on dofetilide-induced arrhythmia were present at the cellular level. Experiments were performed under current clamp control in left ventricular myocytes. Preliminary studies found that only a minority of cells had EADs and DADs when exposed to dofetilide alone (5 µmol/L). In contrast, dofetilide in combination with low external K^+^ (2 mmol/L) resulted in EADs and DADs in the majority of cells. Under these conditions, both EADs and DADs gave rise to triggered APs. The effects of sildenafil are shown in Figure 4D through 4F, where sildenafil (at both 20 nmol/L and 1 µmol/L concentrations) was seen to abolish DADs (*P*=0.03; Figure 4Diii and 4Fii). Under the conditions of these experiments, there was no significant effect on EAD occurrence (Figure 4E and 4Fiii). Furthermore, in 3 cells that showed DADs but not EADs, sildenafil (20 nmol/L) abolished all DADs. Washout of sildenafil led to the return of DADs (Figure 4Div). Because APD is prolonged by the presence of EADs, we have only examined the effect of sildenafil on APD in those cells that did not display EADs. In these 6 cells, consistent with the in vivo findings, there was no effect on APD (Figure 4Fi). Given the effect of sildenafil on DADs, the next series of experiments were directed at understanding its effects on Ca^2+^ waves and Ca^2+^ handling.

### Effect of Sildenafil on Ca^2+^ Cycling

In view of the role of Ca^2+^ in the generation of DADs,^4–6,21,35^ subsequent experiments were designed to determine whether the antiarrhythmic effects of sildenafil were mediated by changes in intracellular Ca^2+^ cycling. Cellular calcium waves were induced in voltage-clamped left ventricular myocytes by raising the external Ca^2+^ concentration to 10 to 15 mmol/L to increase SR Ca^2+^ content (control, 44.3±12.6 µmol/L; high Ca^2+^, 104.4±14.6 µmol/L; *P*=0.004; Figure IV in the Data Supplement). An analysis of the effects of raising external Ca^2+^ on cellular Ca^2+^ handling is presented in the Data Supplement (Table III in the Data Supplement).

**Figure 5.**
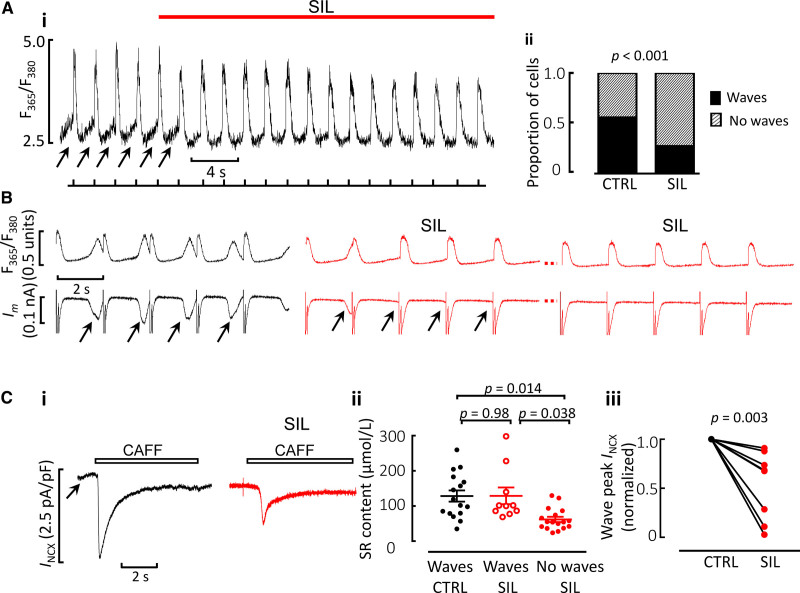
**Sildenafil suppresses Ca^2+^ waves via a reduction in sarcoplasmic reticulum (SR) content.Ai**, Representative trace from a cell stimulated under voltage clamp in 15 mmol/L Ca^2+^ demonstrating the effect of sildenafil (1 µmol/L) on Ca^2+^ wave occurrence. Diastolic Ca^2+^ waves are indicated by arrows. Cells were held at −40 mV with a 100-ms depolarization step to +10 mV. **Aii**, Mean data summarizing the effect of sildenafil on Ca^2+^ waves (36 cells from 20 animals, χ^2^ test). **B**, Paired [Ca^2+^]_i_ and membrane current traces at different time points from a cell under voltage clamp exposed to sildenafil: before sildenafil (**left**), after a 10 s exposure to sildenafil (**middle**), and after 40 s exposure to sildenafil (**right**). **C**, Effect of sildenafil on SR Ca^2+^ content and its relationship to Ca^2+^ waves. **Ci**, Representative paired current recordings during application of caffeine. **Cii**, Mean data summarizing the effect of sildenafil on SR content. **Ciii**, Mean data summarizing the effect of sildenafil on size of waves. For SR content; 16 cells/12 animals (waves in control), 10 cells/10 animals (waves in sildenafil), and 17 cells/10 animals (no waves in sildenafil), unpaired *t* test in all comparisons. For wave peak *I*_NCX_; 8 cells/6 animals, 1 sample *t* test. CAFF indicates caffeine.

As shown in Figure 5Ai, sildenafil reduced Ca^2+^ transient amplitude by 25.6±7.3% (*P*=0.0045; n=22 cells/13 animals). In 12 of 22 cells, sildenafil abolished Ca^2+^ waves (Figure 5Aii; *P*<0.001). In separate, 90 s duration, time control experiments (not shown), waves persisted in all cells studied, and there was no statistical difference in wave properties (n=6 cells/4 animals). In those cells where Ca^2+^ waves remained, sildenafil had 2 effects: (i) delaying the onset of the Ca^2+^ wave relative to the preceding Ca^2+^ transient and (ii) reducing the amplitude of the wave (Figure VC in the Data Supplement) and the resulting peak inward NCX current (Figure 5Ciii). In combination, these effects resulted in a decrease in Ca^2+^ efflux per wave (to 55.2±10.7% of control; *P*=0.003) and per cycle (12.6±4.8 µmol/L per 2-s cycle in control versus 6.5±3.2 sildenafil; *P*=0.016).

### Sildenafil Suppresses Ca2+ Waves by Reducing SR Content Without Altering the Threshold SR Ca^2+^ Content for Ca^2+^ Waves

The next experiments were designed to investigate how sildenafil decreased the occurrence of Ca^2+^ waves. Two possibilities were considered: (i) a reduction of SR Ca^2+^ content and (ii) an increase in the threshold SR Ca^2+^ content for Ca^2+^ wave initiation. In those cells where sildenafil abolished Ca^2+^ waves, there was a decrease of SR Ca^2+^ content (Figure 5Ci and 5Cii, right hand column). Conversely, in those cells continuing to wave in sildenafil, SR Ca^2+^ content (and, therefore, the threshold) was not statistically altered (Figure 5C); however, wave frequency was reduced. A minority of cells (3 cells from 3 animals) did not show Ca^2+^ waves in elevated external Ca^2+^, and in these cells, sildenafil decreased SR Ca^2+^ content (from 58.3±7.1 to 34.0±5.4 µmol/L; *P*=0.033). Thus, we conclude that sildenafil reduces Ca^2+^ wave occurrence by decreasing SR Ca^2+^ content.

### Mechanisms Underpinning the Decrease of SR Ca^2+^ content and Maintenance of Ca^2+^ Flux Balance in Sildenafil

The above results raise 2 questions: (i) what is the mechanism of the reduction in SR Ca^2+^ content and (ii) how does the cell compensate for the loss of Ca^2+^ efflux during Ca^2+^ waves to preserve Ca^2+^ flux balance in sildenafil? We have investigated the following Ca^2+^ handling pathways.

#### L-Type Ca^2+^ Current

**Figure 6.**
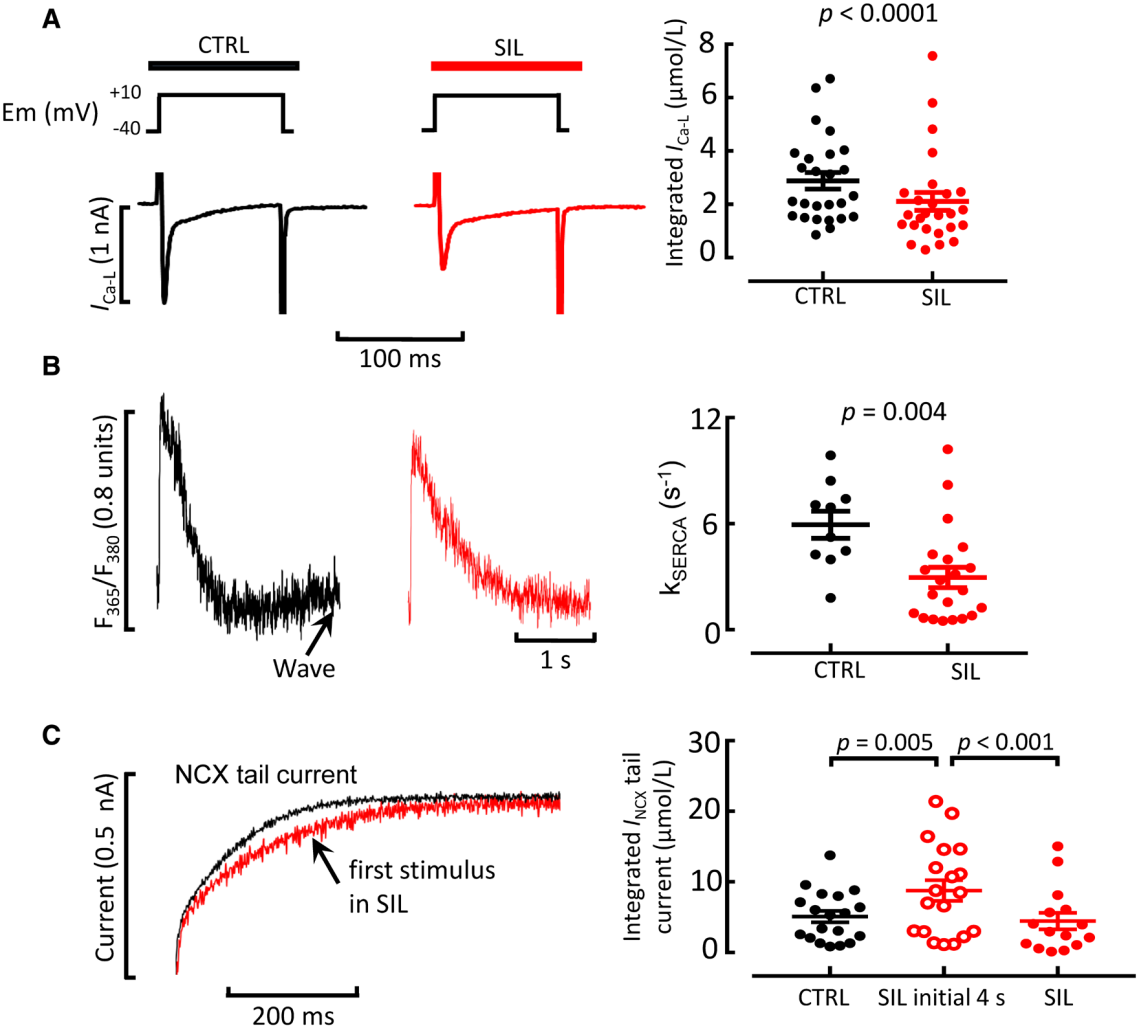
**The effects of sildenafil on sarcolemmal Ca^2+^ fluxes.A**, Effect of sildenafil on *I*_Ca-L_. Representative paired current recordings under voltage clamp (**left**) and paired summary data (**right**). n=26 cells/16 animals. **B**. Effect of sildenafil on SERCA (sarcoplasmic endoplasmic reticulum Ca^2+^ ATPase) activity. Representative Ca^2+^ transients from the same cell (**left**) and unpaired summary data for k_SERCA_ (**right**). Control n=10 cells/10 animals, sildenafil n=21 cells/12 animals. **C**, Applying sildenafil is accompanied by a transient (initial 4 s) increase in the *I*_NCX_ tail current. Representative paired repolarization (NCX [Na^+^-Ca^2+^ exchanger]) tail currents immediately before sildenafil and 4 s after sildenafil (**left**), and summary data (**right**). Experiments performed in 10 to 15 mmol/L Ca^2+^ with and without sildenafil (1 µmol/L). Paired *t* test for all.

Sildenafil decreased peak *I*_Ca-L_ (from 7.76±0.97 to 3.87±0.64 pA/pF; *P*<0.000001; n=28 cells/16 animals; 10–15 mmol/L external Ca^2+^), increased the 90% to 10% inactivation time (from 28.7±1.8 to 34.9±1.9 ms; *P*=0.004), and decreased the integral of *I*_Ca-L_ (from 2.88±0.31 to 2.11±0.34 µmol/L; *P*<0.0001; Figure 6A). However, this (≈0.8 µmol/L) decrease of integrated Ca^2+^ entry is much smaller than the decrease of Ca^2+^ efflux (over each stimulus cycle) caused by the abolition of Ca^2+^ waves (≈7 µmol/L per wave), and additional factors must contribute to maintenance of cellular Ca^2+^ flux balance. In addition, the decreased Ca^2+^ entry is unlikely to explain the reduction in SR Ca^2+^ content since a decrease of L-type Ca^2+^ current does not reduce SR Ca content.^36^

#### Reduced SERCA Function

As illustrated in Figure 6B, sildenafil decreased the rate constant of decay of the systolic Ca^2+^ transient k_SYS_ and the calculated contribution due to SERCA (k_SYS_: 6.5±0.7 s^−1^ in control versus 3.7±0.7 s^−1^ in sildenafil, *P*=0.0012, n=17 cells/11 animals; k_SERCA_: 5.9±0.8 s^−^^1^ in control versus 3.0±0.6 s^−1^ in sildenafil, *P*=0.004). In subsequent experiments (Figure VI in the Data Supplement), we examined whether the sildenafil reduction in SERCA activity could quantitatively account for the effects on Ca^2+^ waves; the irreversible SERCA inhibitor thapsigargin was used to reduce SERCA function. Initial experiments showed that a 1-minute exposure to thapsigargin (5 µmol/L) decreased k_SYS_ from 6.4±0.9 to 3.8±0.6 s^−^^1^, a reduction comparable to that produced by sildenafil (11 cells/7 animals; *P*=0.017). There was no statistically significant effect of thapsigargin on *I*_Ca-L_. Accompanying the reduction in SERCA function, waves were abolished in 6 of 13 cells (*P*=0.007; Figure VI in the Data Supplement, by comparison, waves were abolished in 12 of 22 in sildenafil). In these cells SR Ca^2+^ content was reduced below threshold (to 34.2±7.4 µmol/L; *P*=0.015; n=6–16 cells/4–12 animals). In the remaining 7 cells, which continued to show waves, thapsigargin mimicked sildenafil in reducing the amplitude and delaying the peak of the waves. The mechanism by which the reduced SERCA activity contributes to the reduction of SR Ca^2+^ content is indicated in Figure 6C. Here, the slowed decay of the systolic Ca^2+^ transient promotes increased Ca^2+^ removal from the cell by the NCX. Thus, we conclude that a decrease in SERCA function is a major factor underlying the suppression of Ca^2+^ waves and reduction of SR Ca^2+^ content in sildenafil.

#### Reduced Background Influx

**Figure 7.**
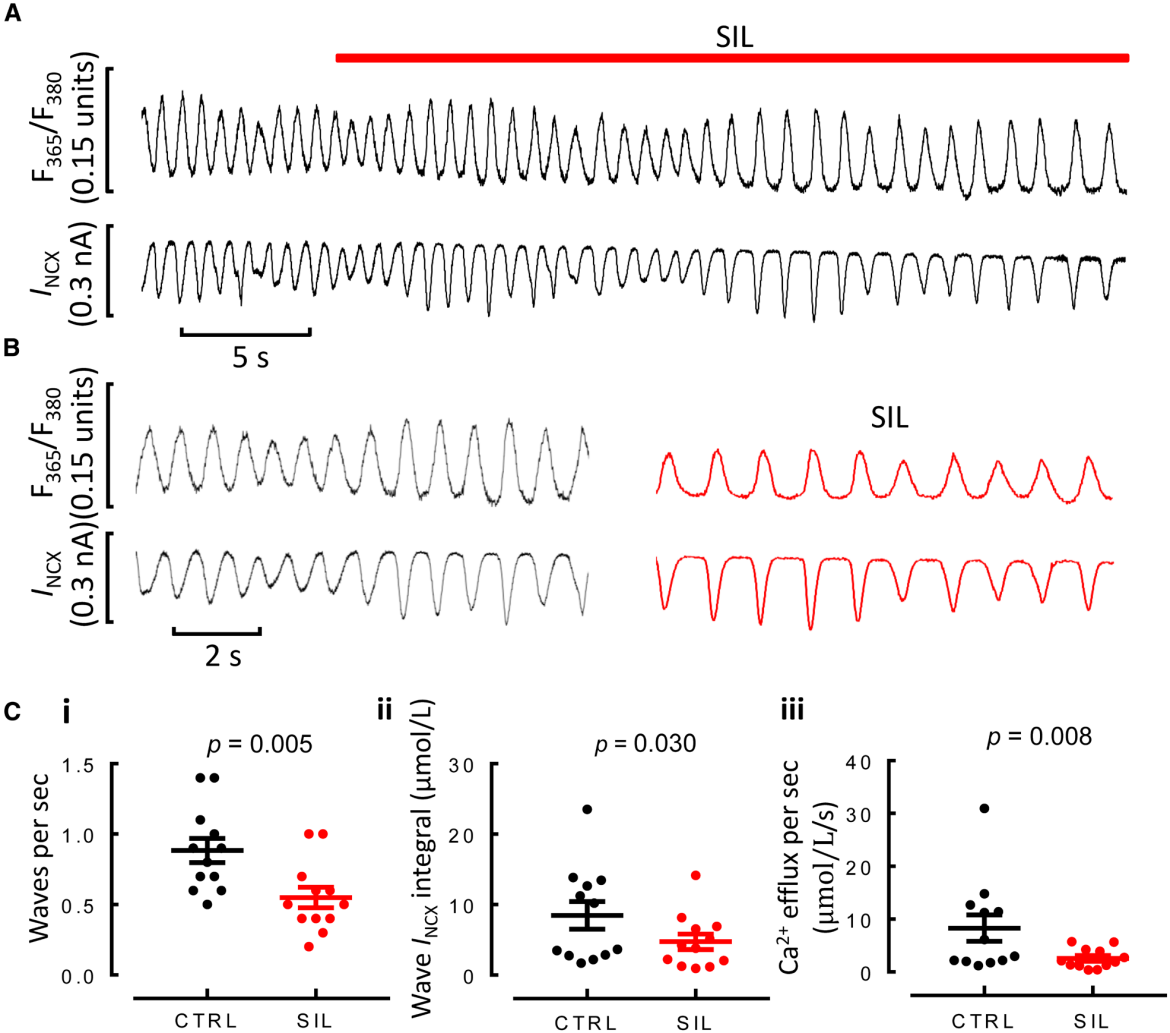
**Sildenafil decreases background Ca^2+^ influx reducing SR Ca^2+^ content.A**, Representative time course of a cell under voltage clamp displaying spontaneous waves, showing the effect of sildenafil on [Ca^2+^]_i_ (**top**) and membrane current (**bottom**). **B**, Comparison between control and sildenafil at steady state. **C**, Summary data showing the effect of sildenafil on wave frequency (**Bi**), wave integral *I*_NCX_ (**Bii**), and Ca^2+^ efflux per second (**Biii**). n=12 cells/7 animals, paired *t* test. Experiments performed in 10 to 15 mmol/L Ca^2+^ with and without sildenafil (1 µmol/L).

Ca^2+^ entry also occurs under resting conditions via a mechanism distinct from *I*_Ca-L_ or *I*_NCX_.^37^ To understand whether sildenafil suppresses waves by modifying this background influx, unstimulated cells were held at the same holding potential as under paced conditions (−40 mV) and spontaneous waves induced with high Ca^2+^ solution (10–15 mmol/L; Figure 7). Under these conditions, background Ca^2+^ influx must equal efflux, and efflux can be estimated as the integral of wave *I*_NCX_ per unit time (analyzed over 10 s). After applying sildenafil, cells were again allowed to return to steady state before a further calculation of background influx was made. Sildenafil decreased wave frequency (Figure 7A and Ci) and decreased *I*_NCX_ on each wave (Figure 7Cii). Together, these resulted in a reduction of Ca^2+^ efflux per unit time (Figure 7Ciii) and, therefore, background influx from 8.3±2.5 µmol/L/s in control to 2.6±0.5 µmol/L/s in sildenafil (*P*=0.008; 12 cells from 7 animals).

In some cells, we examined whether the fects of sildenafil were reversible. Removal of sildenafil led to the reappearance of waves in 4 of 5 cells, and in 2 cells that continued to display Ca^2+^ waves in sildenafil, washout increased the size of waves. Accompanying the return of waves was a reversal of the sildenafil effects on Ca^2+^ cycling, namely an increase in k_SYS_ (6.21±1.29 s^−^^1^ in washout versus 3.10±0.96 sildenafil; *P*=0.003; n=4 cells/3 animals), and an increase in sarcolemmal influx via *I*_Ca-L_ (2.69±0.38 in washout versus 1.79±0.33 µmol/L in sildenafil; *P*=0.047; n=7 cells/4 animals).

### Sildenafil Prevents DAD-Evoked APs

**Figure 8.**
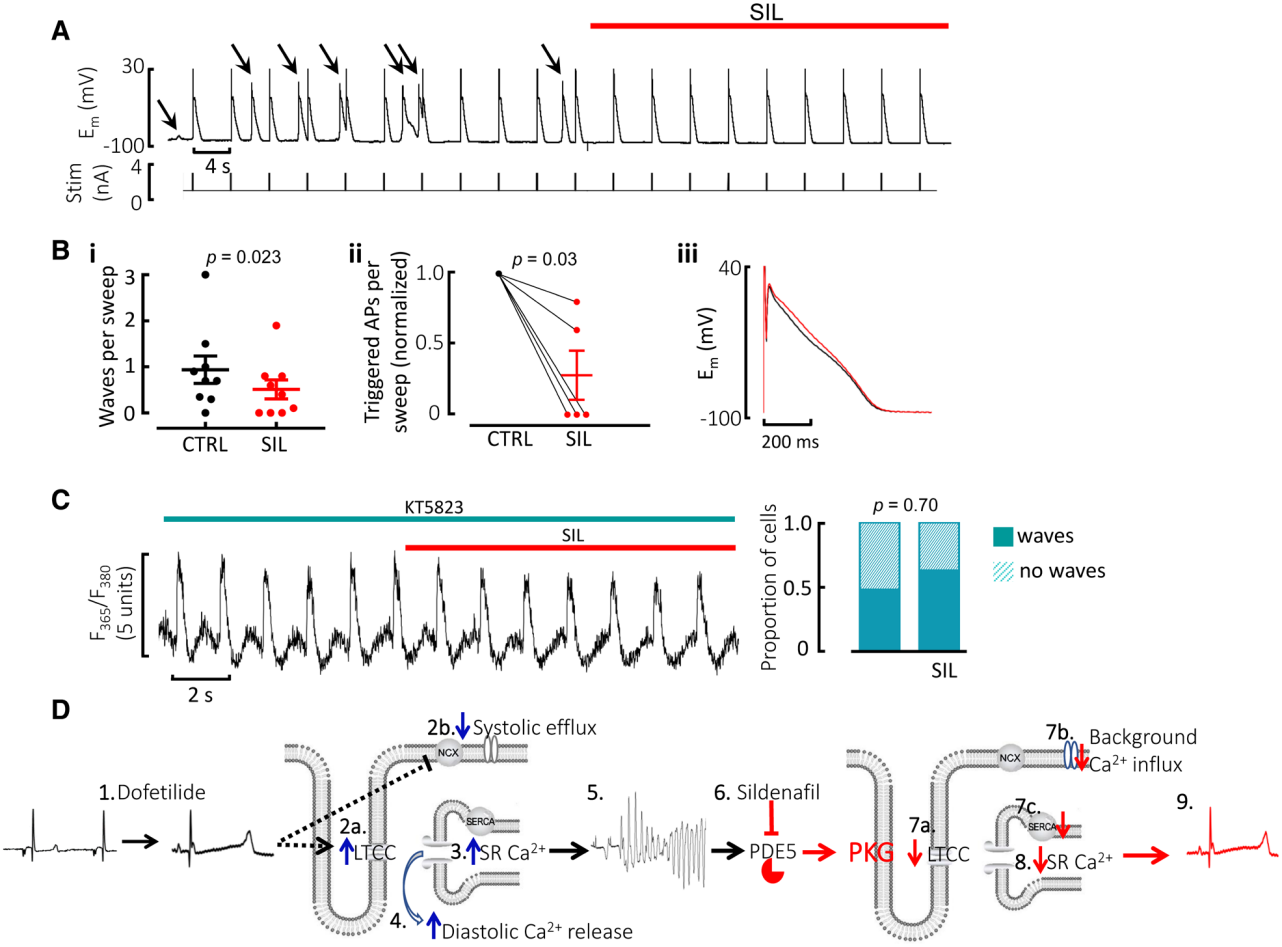
**Mechanism by which sildenafil suppresses triggered action potentials in isolated myocytes.A**, The effect of sildenafil on Ca^2+^ waves is sufficient to prevent triggered action potentials. Representative recording under current clamp of a cell displaying spontaneous waves and triggered action potentials. **B**, Mean summary data of sildenafil effect on waves (**Bi**) and triggered action potentials (**Bii**). For waves, n=9 cells/3 animals. For triggered action potentials, n=5 cells/3 animals, Wilcoxon signed-rank test. **Biii**, Paired recordings of stimulated action potentials before and after sildenafil. K^+^ was reduced to 2 mmol/L and Ca^2+^ elevated to 5 to 7.5 mmol/L to induce Ca^2+^ waves. **C**, Sildenafil suppression of Ca^2+^ waves is lost in the presence of PKG (protein kinase G) inhibitor KT5823. **C**, **Left**, Representative Ca^2+^ recording in a cell displaying waves while being stimulated under voltage clamp. **C**, **Right**, mean paired data of proportion of cells displaying waves. n=13 cells/8 animals, Fisher’s exact test. **D**, Summary diagram. 1, *I*_Kr_ block with dofetilide prolongs the action potential and QT interval, leading to an increase in Ca entry via L-type Ca^2+^ channel (LTCC; 2a) and reducing Ca efflux on NCX (Na^+^-Ca^2+^ exchanger; 2b). In combination, this leads to an increase in sarcoplasmic reticulum (SR) Ca^2+^ content (3), and spontaneous Ca^2+^ release (4), giving rise to afterdepolarizations and ventricular arrhythmias (5). 6, Sildenafil inhibits PDE5 (phosphodiesterase 5) thus activating PKG. PKG-dependent effects include reducing Ca^2+^ influx via the LTCC (7a), reducing background Ca^2+^ influx (7b), and reducing SERCA (sarcoplasmic endoplasmic reticulum Ca^2+^ ATPase; 7c). These combined effects reduce SR Ca^2+^ content below threshold (8), preventing delayed afterdepolarizations and arrhythmias (9).

We next addressed whether the effect of sildenafil on Ca^2+^ waves could reduce afterdepolarizations sufficiently to prevent triggering of arrhythmogenic APs. Cells were paced at 0.25 Hz in current clamp mode using the perforated patch technique. While solutions containing 10 to 15 mmol/L Ca^2+^ were effective at inducing Ca^2+^ waves, we found that the accompanying afterdepolarizations failed to trigger APs (possibly via membrane stabilization).^38^ When external K^+^ was decreased to 2 mmol/L, Ca^2+^ waves were initiated at a lower external Ca^2+^ (5–7.5 mmol/L); the resulting afterdepolarizations now triggered APs (Figure 8A). Under these conditions, sildenafil decreased both the frequency of Ca^2+^ waves and the resulting triggered APs (Figure 8B) but had no significant effect on APD (Figure 8Biii). Thus, we conclude that the sildenafil effect on waves has clinical relevance in preventing triggering of APs both in single cells and in vivo.

### PKG Inhibition Abolishes the Protective Effect of Sildenafil on Ca^2+^ Waves

To determine whether the sildenafil reduction in wave propensity depends on PKG, control cells were preincubated for >30 minutes with the PKG inhibitor KT5823 (1 µmol/L) and the effects of sildenafil on waves examined (Figure 8C). Under PKG inhibition, sildenafil failed to suppress waves in all 6 cells tested. In addition, sildenafil had no significant effect on the peak or integral of ICa-L. We, therefore, conclude that the protective effect of sildenafil on waves is PKG dependent.

## Discussion

We have investigated the effects of sildenafil on arrhythmias both in vivo and in isolated myocytes. As summarized in Figure 8D, there were 5 major findings. (1) Sildenafil suppresses TdP by reducing both the frequency of PVCs and the probability of a PVC initiating TdP. (2) The protection against TdP afforded by sildenafil occurs due to later timing of PVCs such that they occur after resolution of the T wave, protecting from an R on T effect. (3) In isolated myocytes, PDE5 inhibition suppresses Ca^2+^ waves and triggered APs by reducing SR content. (4) The reduction in SR content results from a decreased SERCA activity with additional contributions from reduced background Ca^2+^ entry. (5) The effects of sildenafil on Ca^2+^ waves are PKG dependent.

### Comparison With Previous Work

An antiarrhythmic effect has been demonstrated previously when sildenafil was administered before myocardial ischemia, although the underlying mechanisms were not elucidated. In those studies, administration of sildenafil ≈20 hours before canine acute coronary ischemia and reperfusion reduced incidence of PVCs, VT, and VF,^39^ while in isolated rat hearts, sildenafil decreased VF.^40^ It is possible that the reduction in arrhythmias was not a direct effect of sildenafil but, rather, secondary to an ischemic preconditioning effect since, in the former, sildenafil serum concentrations are expected to have decayed to low levels at the time of arrhythmia.^41^ In the latter, sildenafil substantially reduced infarct size. A number of studies have now implicated PDE5 inhibition in ischemic pre- and postconditioning, cardioprotection and ischemia-reperfusion injury,^42^ reducing infarct size, apoptosis, and postinfarct remodeling, and via a pathway involving opening of BK channels.^43,44^ Such effects are likely contributors in explaining reduced arrhythmias in these contexts. To our knowledge, this article is the first to demonstrate direct antiarrhythmic actions of PDE5 inhibition. While we used a dofetilide-induced model of QT prolongation and ventricular arrhythmia, the cellular mechanisms underlying the antiarrhythmic effects of PDE5 inhibition suggest it may additionally hold antiarrhythmic potential in other conditions causing Ca^2+^-dependent arrhythmia, such as heart failure (HF), catecholaminergic polymorphic ventricular tachycardia, and digitalis toxicity.

### Cellular Mechanisms of the Antiarrhythmic Effects of Sildenafil

PDE5 inhibition reduced *I*_Ca-L_ as noted previously.^45^ While it is conceivable that the modest decrease in *I*_Ca-L_ could contribute to the antiarrhythmic effect of sildenafil, it does not appear to be the predominating mechanism. Some previous work has demonstrated that pharmacological block of L-type Ca channels suppresses EADs, but the dose used also decreased APD_90_.^46–48^ In our experiments and a previous study,^45^ sildenafil did not change APD. Additionally, sildenafil also slowed the inactivation of *I*_Ca-L_, presumably because of the smaller Ca^2+^ transients reducing Ca^2+^-dependent inhibition. Given these opposing actions, the overall effect of sildenafil on EADs is difficult to predict. Furthermore the decrease of *I*_Ca-L_ would not be expected to decrease SR Ca content^36^ and, as such, would not be antiarrhythmogenic. It should also be noted that, although *I*_Kr_ reduction is a trigger for EADs and sildenafil was found to suppress EADs in vivo, our cellular findings using elevated external Ca^2+^ or reduced external K^+^ suggest that arrhythmias appear to be most dependent on DADs. Specifically, it is the abolition of the DAD that is associated with the antiarrhythmogenic effects of sildenafil. We cannot, however, exclude the possibility that abolition of EADs also contributes to the removal of arrhythmias in vivo. Finally, the abolition by sildenafil of EADs in vivo may contribute to decreasing cell and, therefore, SR Ca^2+^ loading, either directly by stopping reactivation of the L-type Ca current or, indirectly, by removing the effect of these depolarizations to decrease the activity of NCX.

It is perhaps surprising that the decrease of both the amplitude and integral of the L-type Ca current (Figure 6) is not associated with an effect on APD. In this context, we can only speculate that the slowed inactivation and consequent increase of inward current at late times during the AP plateau may result in sildenafil increasing APD.

Our experiments using thapsigargin (Figure VI in the Data Supplement) show that the degree of inhibition of SERCA by sildenafil is sufficient to suppress Ca^2+^ waves in the majority of cells indicating a major role for reduced SR Ca^2+^ content. In those cells continuing to display waves, the decrease in SERCA prolongs the time taken for the SR to reach threshold, thus delaying onset of the wave. Plausibly, this may explain the delayed PVCs and reduction in R on T events observed in sildenafil. Our experiments also point to another SERCA-independent mechanism; sildenafil reduces background Ca^2+^ entry, which is expected to decrease SR Ca^2+^ content and Ca^2+^ waves.^15^ However, we are unable to quantify background Ca^2+^ entry without raising external Ca^2+^, and, therefore, the contribution to the effects of sildenafil under more physiological conditions remains to be determined. Our finding that inhibiting SERCA suppresses Ca^2+^ waves and thence arrhythmias is in agreement with previous work where SR Ca^2+^ content is reduced below a threshold level.^49^ However, it has also been reported that decreasing SERCA can allow Ca^2+^ waves to propagate more easily^50^ and, correspondingly, increasing SERCA function can prevent Ca^2+^-dependent arrhythmias in the setting of severe SR leak, by restricting SR Ca^2+^ release to miniwaves and Ca^2+^ sparks, thus preventing propagation of cell-wide waves.^51^ In scenarios where SERCA inhibition is insufficient to reduce SR content below threshold, it is conceivable that it could aggravate arrhythmias by organizing cell-wide Ca^2+^ waves, and it will be important, therefore, to test the role of sildenafil under these conditions as well.

Our findings raise several important questions. First, while we have characterized the mechanistic components underlying antiarrhythmic effects of sildenafil, the signaling pathways controlling these events require further exploration. The process appears to be PKG-dependent given that the sildenafil suppression of waves was abolished by KT5823. Exactly how manipulation of the cGMP-PKG axis reduces SR Ca^2+^ content is an important unanswered question. Indeed this is at odds with previous reports of PKG increasing SERCA function via phosphorylation of PLB (phospholamban).^52^ One possibility is that PKG can activate protein phosphatase 1,^53,54^ which could lead to a dephosphorylation of phospholamban and reduce SERCA activity. In our experiments, sildenafil also decreased background Ca^2+^ influx. A background Ca^2+^ influx has been reported before in cardiac myocytes and is sensitive to gadolinium.^37,55^ Transient Receptor Potential (TRP) channels are expressed in cardiac myocytes, are inhibited by gadolinium, and PKG, which is activated by PDE5 inhibition, has been shown to acutely decrease Ca^2+^ entry via TRPC6 inhibition in rat neonatal cardiac myocytes.^56^ Thus, this appears to be a plausible molecular mechanism for the sildenafil decrease in background influx and by changing cytosolic Ca^2+^ and activation of CaMKII, could conceivably modify PLB phosphorylation.

Given the potential roles for cross talk effects between cGMP and the cAMP-PKA (protein kinase A) axis, whether the antiarrhythmic effects of PDE5 inhibition extend to catecholamine-induced arrhythmia is an important consideration in future studies. While Lee et al^23^ demonstrated that sildenafil suppressed β-adrenergic stimulation of contractility in mouse hearts via PKG phosphorylation of cTnI (cardiac troponin I), the effect PDE5 inhibition has on Ca^2+^ cycling under these conditions is difficult to predict given that cGMP has the capacity to inhibit PDE3 and thus has the potential to increase both *I*_Ca-L_ and k_SERCA_.

While sildenafil has selectivity for PDE5, the concentrations used in this study (1 µmol/L and 20 nmol/L) it may to a lesser extent also inhibit other PDEs including PDE1, PDE6, and PDE11.^57^ Effects on PDE6 and PDE11 are unlikely to be relevant as these are not expressed in the heart.^58,59^ Expression of PDE1 has been confirmed in the heart and has been implicated in pathological hypertrophy.^60^ Given that sildenafil has an IC50 (the half maximal inhibitory concentration) for PDE1 of 350 nmol/L,^57^ sildenafil at 1 µmol/L is expected to cause substantial inhibition. Nevertheless, the effects of sildenafil were also reproduced at 20 nmol/L (Figure 4D through 4F; Figure IX in the Data Supplement) where inhibition of PDE1 is expected to be minimized. During in vivo experiments, serum and myocardial concentrations of sildenafil were not determined following administration of intravenous sildenafil, and as such, we cannot be certain about the degree of selectivity in vivo. The dose used (10 mg) was, however, similar to that used clinically (see below).

### Clinical Relevance

Accompanying sildenafil suppression of waves was a reduction in the amplitude of the Ca^2+^ transient by reduced SR Ca^2+^ content and decreased trigger for SR release by *I*_Ca-L_, which would be expected to decrease contractility. In addition, PDE5 inhibitors reduce arterial BP. These are important clinical considerations given that frequently encountered proarrhythmic states are accompanied by impaired ventricular systolic function (eg, heart failure, myocardial infarction) and hemodyamic disturbance. Paradoxically, however, PDE5 inhibitors appear to improve contractile function in clinical and preclinical models of systolic HF and in animal models of myocardial ischemia (MI). In HF, PDE5 inhibitors have neutral effects when administered acutely and overall positive effects on ventricular function following chronic treatment.^61–65^ After MI, PDE5 inhibitors appear to improve contractile function, an effect that may relate to infarct size reduction and preventing adverse remodeling.^66^ While there is reason to be cautious when acutely administering sildenafil in patients with poor contractile function and low arterial BP, the doses used in our in vivo experiments are comparable (even allowing for the sheep weighing half of a human) to those safely administered in humans for pulmonary hypertension and erectile dysfunction^67–69^ and in clinical trials of heart failure.^65^ Furthermore, while its use as an acute antiarrhythmic has never been tested in humans, it is conceivable that its acute negative inotropic and hypotensive effects may be outweighed by acute antiarrhythmic and longer term contractile remodeling effects.^62,70^ One noteworthy finding in our observations of cells showing Ca^2+^ waves is that although Ca^2+^ transient amplitude is reduced, this effect is relatively modest (−26%), yet abolishing waves is expected to improve overall contractile performance by enhancing diastolic performance.^71,72^ Nevertheless, how PDE5 inhibition induces remodeling and improves contractile function in chronic treatment, to overcome acute negative inotropic effects, is an important area for further investigation.

In conclusion, we have demonstrated that PDE5 inhibition with sildenafil acutely suppresses Ca^2+^-dependent triggered arrhythmias in vivo via the suppression of Ca^2+^ waves in cardiac myocytes. Mechanistically, this is achieved via an acute reduction in SERCA function, as well as decreased background Ca^2+^ entry, and depends on a signaling pathway involving PKG. We propose the described effects are both directly clinically relevant and highlight a novel mechanism of arrhythmia suppression not reported previously.

## Sources of Funding

This work was supported by grants from the British Heart Foundation (FS/15/28/31476, FS/12/57/29717, CH/2000004/12801, FS/12/34/29565, and PG/10/89/28630) and the Medical Research Council (MR/K501211/1). D.C. Hutchings and C.M. Pearman were supported by clinical lectureships from the National Institute for Heath Research (NIHR).

## Disclosures

None.

## Supplemental Materials

Expanded Materials and Methods

Data Supplement Figures I–X

Data Supplement Tables I–V

References ^73–79^

Major Resources Table

## Supplementary Material



## Novelty and Significance

### What Is Known?

- Perturbations to Ca^2+^ cycling contribute to arrhythmias in long QT syndromes.
- Incidental use of PDE5 (phosphodiesterase 5) inhibitors is associated with a reduction in post-myocardial infarction mortality in type II diabetes and potential antiarrhythmic effects are unknown.
- PDE5 inhibitors activate cGMP-PKG (protein kinase G) signaling, which can affect Ca^2+^ cycling; however, their effects on Ca^2+^-dependent arrhythmias have not been investigated.
