# Supplementary material for: PDE5 Inhibition Suppresses Ventricular Arrhythmias by Reducing SR Ca2+ Content
Source: Circ Res. 2021 Jul 12;129(6):650–65. doi: 10.1161/CIRCRESAHA.121.318473 (PMC8409902; doi:10.1161/CIRCRESAHA.121.318473)
Supplement: Supplementary file 1 [file res-129-650-s001.pdf]

Supplemental material

Methods

Sheep were group housed (3-5 animals per pen) using a 12:12 hour light:dark cycle, at 19-21°C, received *ad libatum* access to drinking water and were fed hay and ruminant concentrate. No animals were excluded from the study.

Isolation of ventricular myocytes. Following intravenous heparinization (10,000 iu) to inhibit coagulation of coronary vessels, animals were euthanased with pentobarbitone (200mg/kg). The heart was rapidly excised and rinsed in a Ca^{2+}-free solution at room temperature, and ventricles separated from atria. The left anterior descending coronary artery was cannulated and corresponding territory of myocardium perfused with Ca^{2+}-free isolation solution at 37°C for 10mins at 25 ml/min. Following this, an enzyme solution containing type 2 collagenase (0.1 mg/ml, Worthington Biochemical Corporation, NJ, USA), type XIV protease (0.02 mg/ml, Sigma Aldrich, UK), and Ca^{2+} (100μmol/L) was perfused for 6-11 mins. After appropriate digestion, the perfusion was switched to a taurine-containing solution and perfused for a further 20mins. An ~ 2 x 3 cm block of tissue was cut from the mid-myocardial layer of the anterior wall of the left ventricle, finely minced with scissors, then gently triturated with a plastic pipette and filtered through a fine (200 μm) nylon mesh. Solutions were made with milli-Q grade water. All experiments were performed within 12 hrs of isolation. The Ca^{2+}-free solution contained (in mmol/L): NaCl, 134; glucose, 11; HEPES, 10; 2,3-butanedione monoxime (BDM), 10; KCl, 4; $MgSO_4$, 1.2; Na_2HPO_4, 1.2; 0.5 mg/ml bovine serum albumin (fatty acid free, BSA). pH 7.34 with NaOH. The taurine solution contained (in mmol/L): NaCl, 113, taurine, 50; glucose, 11; HEPES, 10; BDM, 10; KCl, 4; $MgSO_4$, 1.2; Na_2HPO_4, 1.2; $CaCl_2$, 0.1; and 0.5 mg/ml BSA. pH 7.34 with NaOH. The Tyrodes' solution contained (in mmol/L): NaCl, 140; glucose, 10; HEPES, 10; KCl, 4; $CaCl_2$, 1.8; $MgCl_2$, 1; pH 7.34 with NaOH.

Whole cell voltage clamp with series resistance compensation. Membrane currents were controlled using the whole cell voltage clamp technique. Following rupture of the patch, access resistance was compensated using the switch clamp facility of the Axoclamp-2B voltage clamp amplifier (Axon instruments, Union City, CA, USA). Cells were superfused with (in mmol/L): NaCl; 140, KCl; 4.0, $MgCl_2$; 1, HEPES; 10, glucose; 10, $CaCl_2$; 1.8, Probenecid; 2, 4-aminopyridine; 5, $BaCl_2$; 0.1, pH 7.34 with NaOH. Electrodes (3 – 4MΩ resistance) were filled with (in mmol/L): CsCl; 118, $MgCl_2$; 4.0, $CaCl_2$; 0.28, Sodium phosphocreatine; 3, HEPES; 10, CsEGTA; 0.02, Na_2ATP; 3.1, Na_2GTP; 0.42, pH 7.2 with CsOH. Cs^+-salts were used instead of K^+ to block outward K^+ currents.

Measurement of $[Ca^{2+}]_i$. Intracellular Ca^{2+} was measured with epifluorescence microscopy using the pentapotassium salt of the ratiometric dye Fura-2, loaded via the patch pipette (100 μmol/L, Invitrogen).[1] Changes in $[Ca^{2+}]_i$ were determined by the degree of Fura-2 excitation at λs 365 and 380 nm and emission measured at 510 nm. The $[Ca^{2+}]_i$ relates to the ratio of light emitted at 365 nm excitation to that emitted at 380 nm excitation, after subtracting background fluorescence.

Quantification of sarcolemmal Ca^{2+} fluxes and SR content. Cell capacitance was measured by applying a short 10 mV hyperpolarizing pulse, and cell volume determined using a capacitance to volume conversion factor of 5.39 pF/pL to allow quantification of sarcolemmal Ca^{2+} fluxes.[2] In all paced experiments under voltage clamp the rate was 0.5 Hz. During voltage-clamp pacing cells were held at a 'resting' E_m of –40 mV and underwent a 100 ms depolarization to +10 mV to initiate $I_{Ca\text{-}L}$. Systolic sarcolemmal Ca^{2+} influx was determined by integration of $I_{Ca\text{-}L}$, and peak $I_{Ca\text{-}L}$ was determined as the point of maximal inward current relative to a zeroed baseline. The efflux of Ca^{2+} via I_{NCX} associated with the Ca^{2+} transient was quantified by integration of the NCX current immediately after repolarization. SR content was measured at -40 mV via the rapid application of 10 mmol/L caffeine (Sigma-Aldrich, UK) to release Ca^{2+} from the SR, and integration of the ensuing inward NCX current (I_{NCX}).[3, 4] In cells displaying Ca^{2+} waves, threshold SR content was determined as the integral of wave I_{NCX} plus the integral of the caffeine-induced I_{NCX}. In these cells, caffeine application resulted in a maintained increase of $[Ca^{2+}]_i$ above the pre-caffeine baseline value as previously reported.[5] Because the elevated $[Ca^{2+}]_i$ leads to a maintained inward (I_{NCX}) current, the caffeine-induced I_{NCX} was integrated to this new baseline.

Activity of $[Ca^{2+}]_i$ removal mechanisms. Rate constants for Ca^{2+} removal from the cytosol (k_{SYS}) and Ca^{2+} extrusion from the cell (k_{CAFF}) were determined by fitting a first order exponential function to the decay phases of the systolic and caffeine-induced Ca^{2+} transients, respectively. k_{SYS} represents the summation of both Ca^{2+} extrusion from the cell (via NCX, PMCA, and mitochondrial uptake; I_{MCU}) and uptake into the SR (via SERCA), while k_{CAFF} reflects Ca^{2+} extrusion from the cell alone (via NCX and to a lesser extent 'slow efflux' mechanisms of PMCA and I_{MCU}). Therefore the rate constant of uptake into the SR, K_{SERCA}, is calculated using the equation $k_{SERCA} = k_{SYS} - k_{CAFF}$.[6]

In vivo studies. A five-lead electrocardiogram (EMKA technologies, Paris, France) and endocardial electrograms were digitized to a personal computer and analyzed in LabChart 7 (AD Instruments). Monophasic action potential duration was analysed using custom-written software[7]. In determining the QT interval, the end of the T wave was defined using the tangent method as previously described.[8, 9]

Beat variability of the QT interval (BVQTI) was calculated using the following equation[10]:

$$BVQTI = \Sigma|QT_{n+1} - QT_n|/[30 \times \sqrt{2}]$$

Supplemental Figures

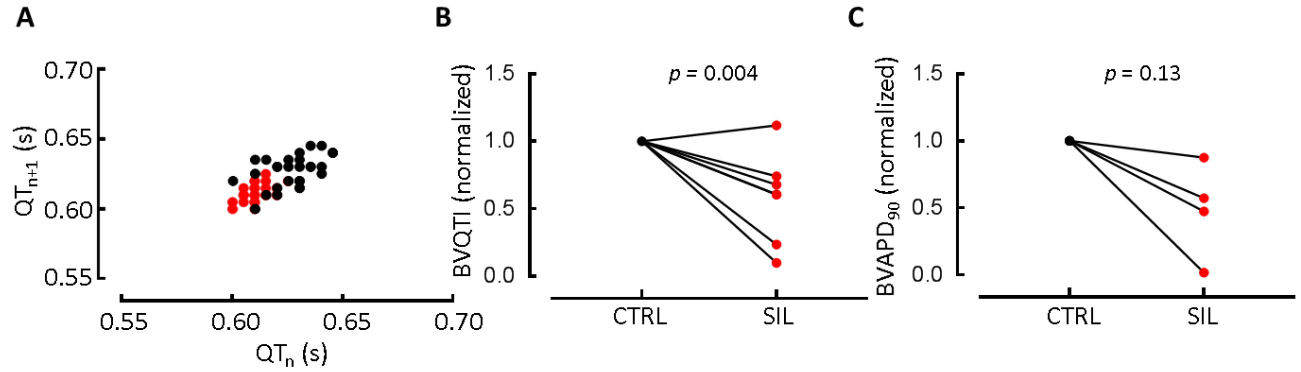

Supplemental Figure I. Sildenafil reduces beat-to-beat variability of repolarization. A. Representative Poincare plot of sequential QT intervals from a single animal before (black) and after sildenafil (red). **B & C.** Summary data of sildenafil effect on beat variability of QT interval index and *in vivo* beat variability of action potential duration, respectively. For BVQTI, *N* = 6 animals, one-sample t test. For $BVAPD_{90}$, *N* = 4 animals, Wilcoxon matched pairs signed rank test.

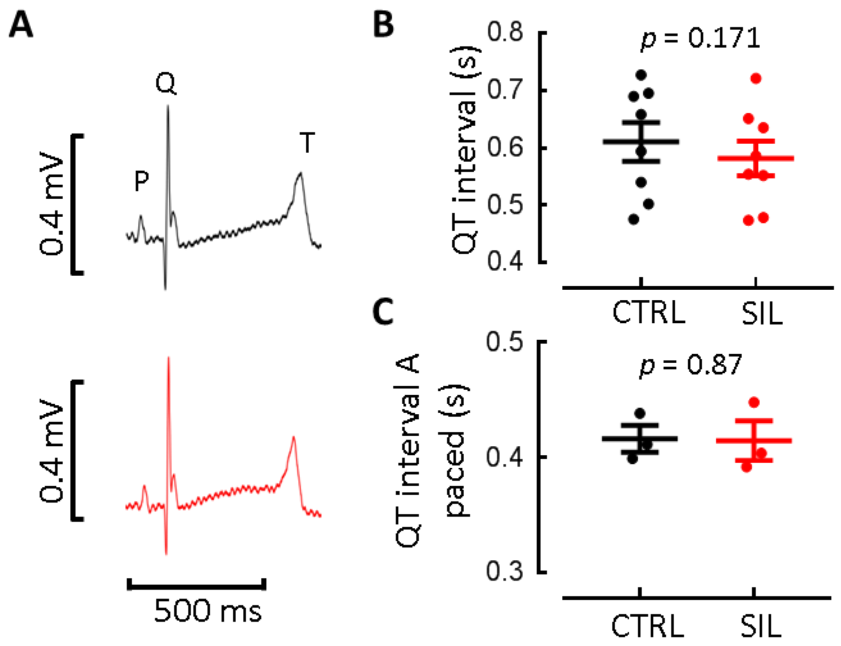

Supplemental Figure II. Reduction in 'R on T' events in sildenafil is not due to changes in the QT interval. A. Representative paired surface ECG recordings in control (upper) and sildenafil (lower). **B & C.** Summary data of QT interval in sinus rhythm (*N* = 8, paired t test) and during atrial pacing above the sinus rate (*N* = 3, Wilcoxon matched pairs signed rank test).

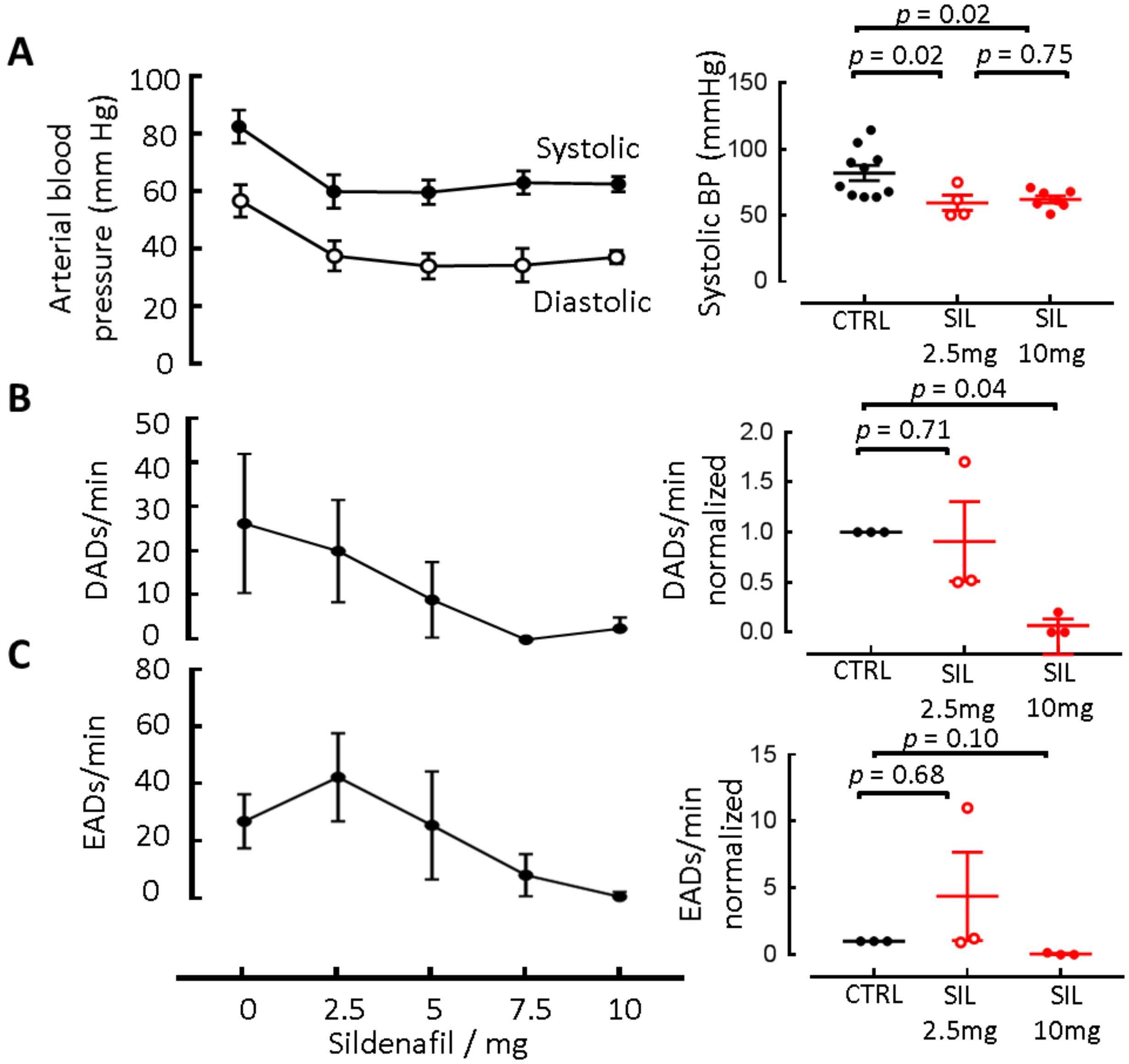

Supplemental Fig III. Antiarrhythmic effects of sildenafil are independent of changes in systemic arterial afterload. A. Effect of incremental doses of sildenafil on arterial pressure (left) and summary data (right). *N* = 4 - 10 animals, Kruskal-Wallis test. **B** & **C**. Effect of incremental sildenafil doses on DADs and EADs, respectively (left) , and summary data (right). Paired data from *N* = 3 animals, Friedman test.

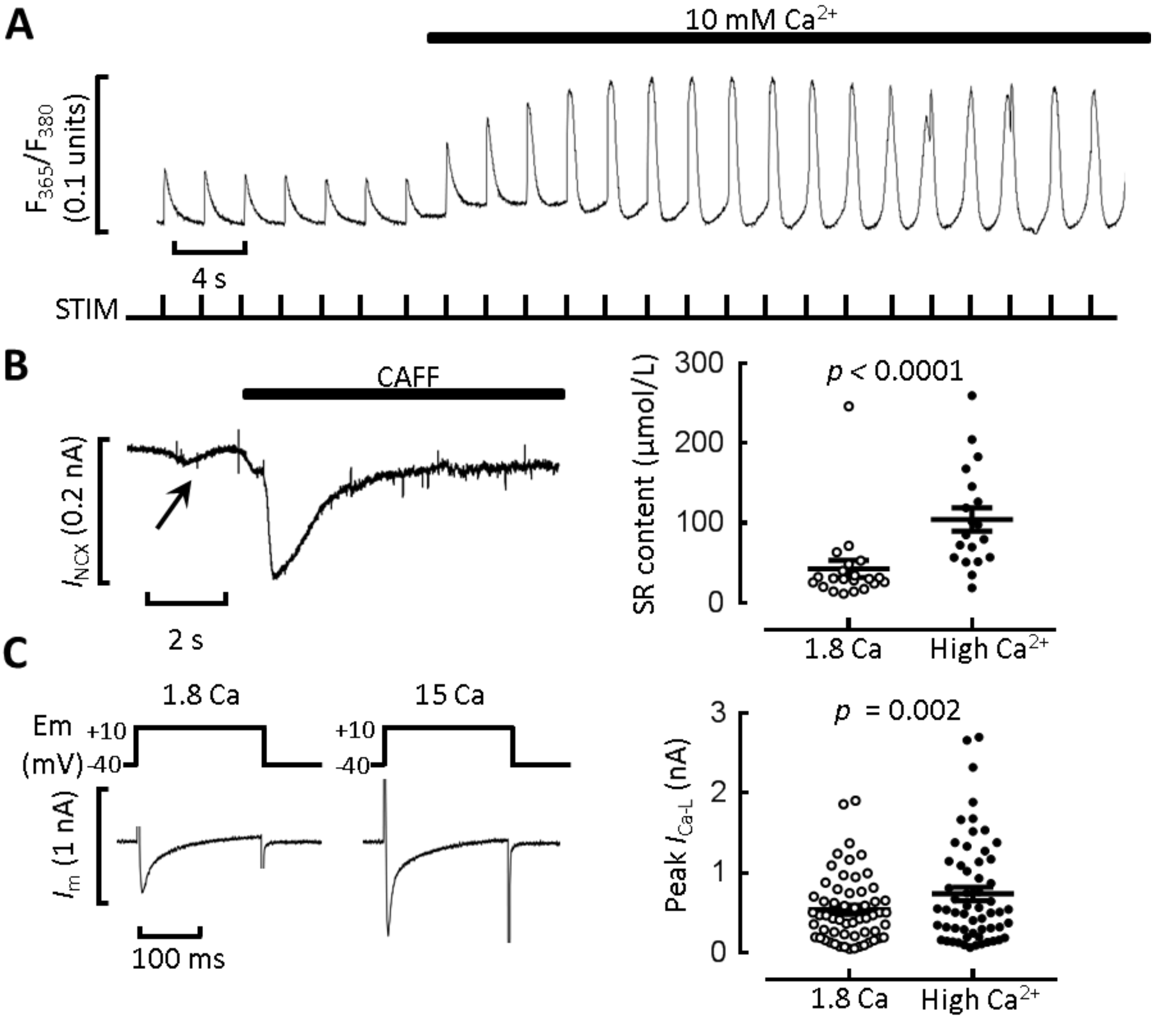

Supplemental Fig IV. Elevating external Ca^{2+} to 10-15mmol/L increases SR content and induces diastolic waves. A. Representative $[Ca^{2+}]_i$ recording in a cell exposed to elevated external Ca^{2+}. **B**. Representative membrane current recording (left) of a cell in high Ca^{2+} (15 mmol/L) exposed to caffeine immediately following a wave (wave indicated by arrow), and summary data (right) showing effect of high Ca^{2+} on SR content. *n* = 19 - 21 cells / *N* = 12 – 16 animals, Mann Whitney U test. **C**. Representative paired L-type current recordings (left) before and during exposure to high Ca^{2+} solution (10 – 15 mmol/L) and summary data (right). Paired data from *n* = 58 cells / *N* = 31 animals, paired t test (on cells).

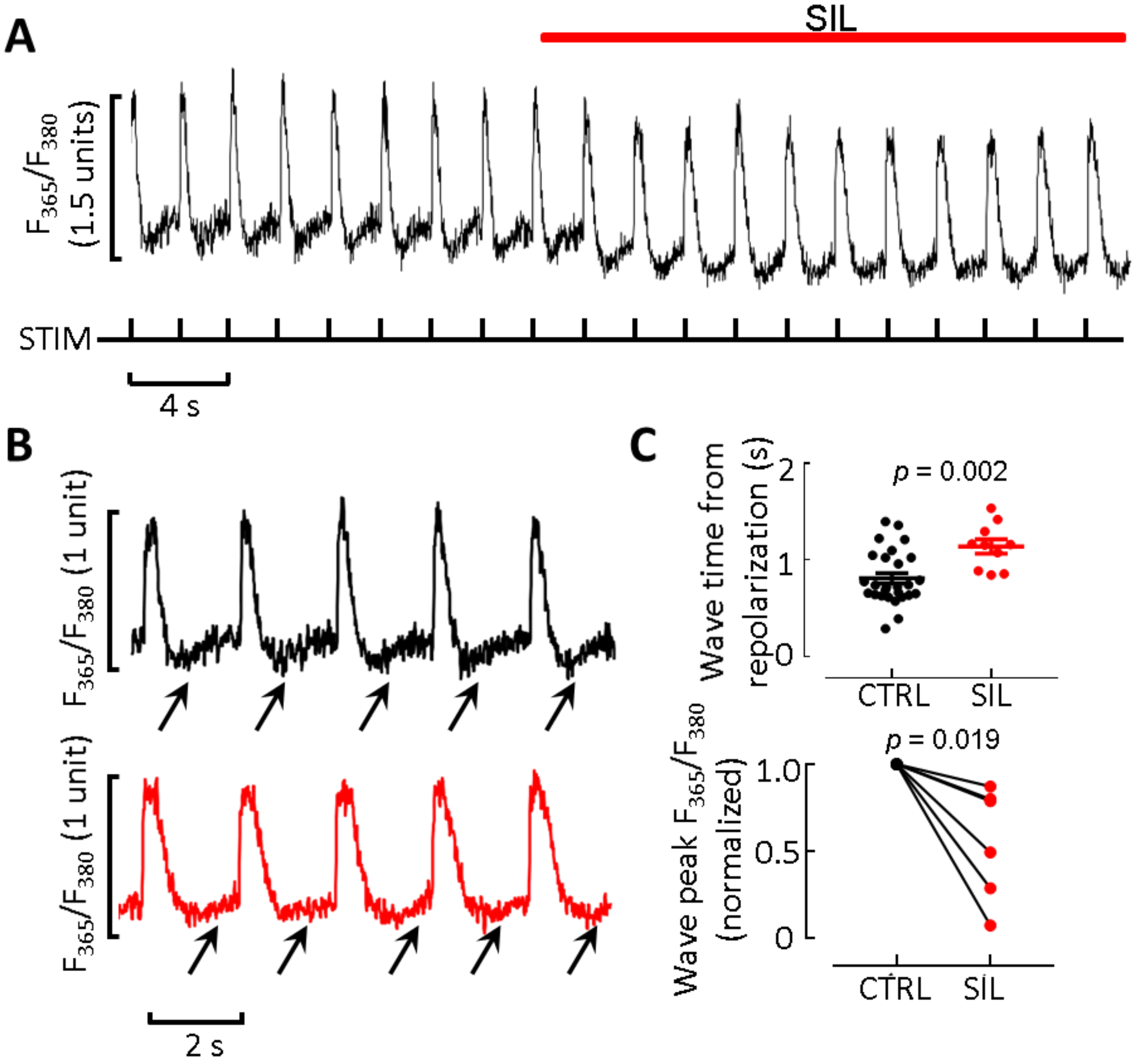

Supplemental Figure V. In cells continuing to display waves in sildenafil, wave size is reduced and their onset is delayed. **A**. Representative $[Ca^{2+}]_i$ recordings from a cell under voltage clamp displaying waves in sildenafil. **B**. Expanded traces immediately prior to sildenafil (upper) and during exposure to sildenafil (lower). Onset of waves is indicated by arrows. **C**. Summary data showing the effect of sildenafil on wave time from repolarization (upper) and wave peak Ca^{2+} amplitude (lower). For timing from repolarization *n* = 10 - 27 cells / *N* = 6 – 16 animals, unpaired t test. For wave peak Ca^{2+} amplitude, paired data from *n* = 6 cells / *N* = 5 animals, one-sample t-test.

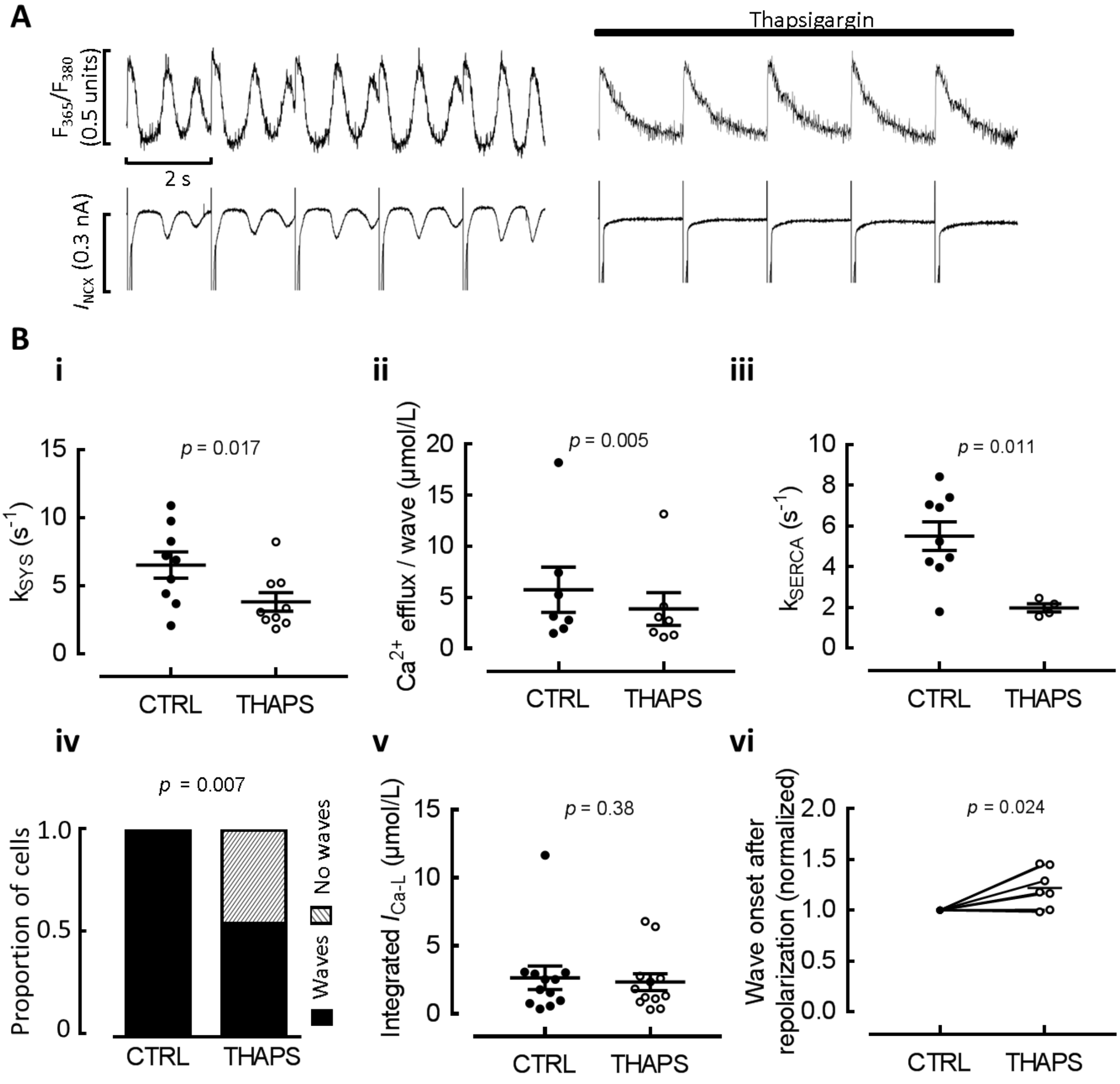

Supplemental Figure VI. SERCA inhibition with thapsigargin mimics sildenafil suppression of waves. A. Upper trace; Representative paired $[Ca^{2+}]_i$ and membrane current recordings showing effects of thapsigargin in 15 mmol/L external Ca^{2+}. **B**. Mean data showing effect of thapsigargin on Ca^{2+} handling, suppression of waves, and properties of waves. For k_{SYS}, N = 9 cells / 8 animals, paired t test. For Ca^{2+} efflux / wave n = 7 cells / N = 6 animals, paired t test. For K_{SERCA} N = 4 – 9 cells / 4 – 9 animals, Mann Whitney test. For proportion cells waving n = 13 cells / N = 9 animals, Chi Square test. For integrated $I_{Ca\text{-}L}$ n = 12 cells / N = 8 animals, paired t test. For time from repolarization n = 7 cells / N = 5 animals, one-sample t test.

.

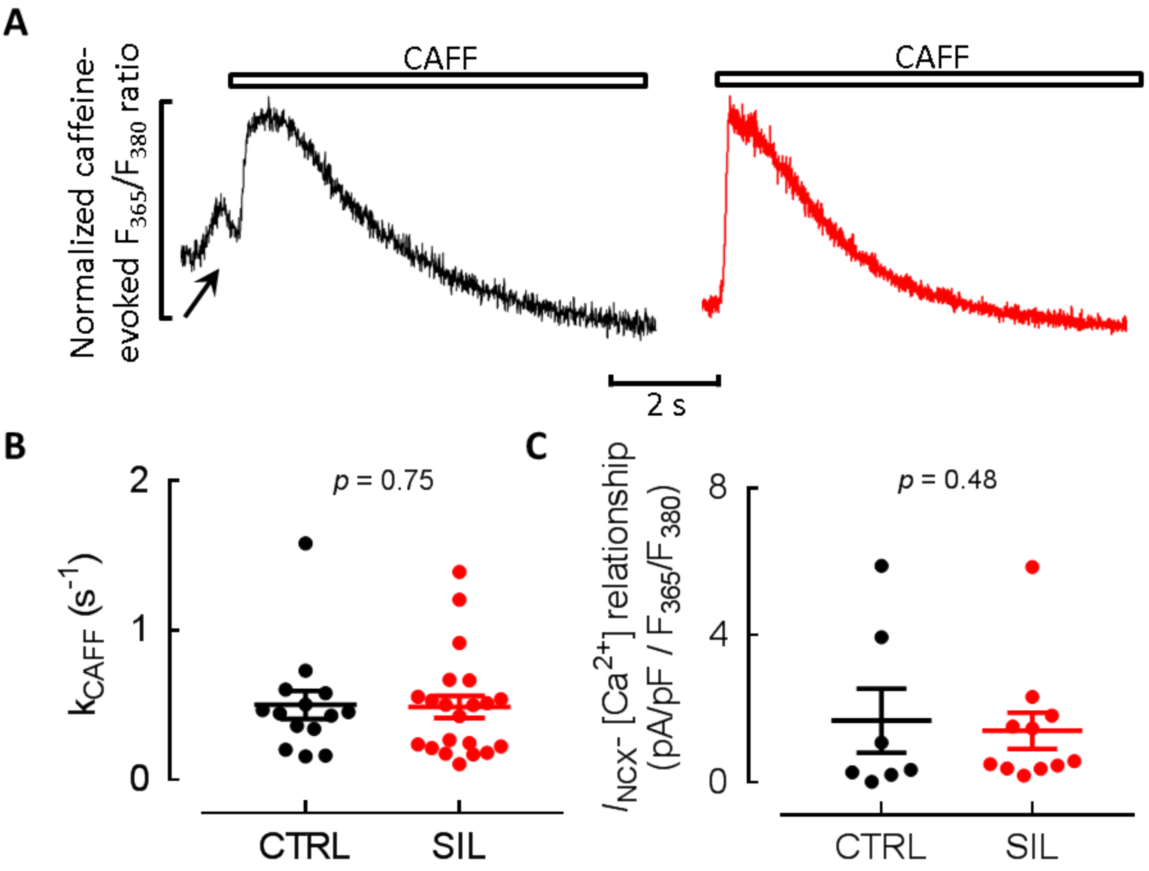

Supplemental figure VII. Sildenafil does not modify sarcolemmal extrusion mechanisms in high external Ca^{2+}. **A.** Representative caffeine-evoked Ca^{2+} transients (normalized). Arrow indicates a wave immediately preceding the application of caffeine. **B.** Mean data summarizing rate of decay of caffeine-evoked transients (k_{CAFF}). *n* = 14 - 21 cells / *N* = 12 - 12 animals, unpaired t test. **C.** Mean data summarizing the relationship between I_{NCX} and $[Ca^{2+}]_i$ (F_{365}/F_{380} ratio). Absolute values are plotted. *n* = 7 - 11 cells / *N* = 7 - 7 animals, Mann Whitney U test.

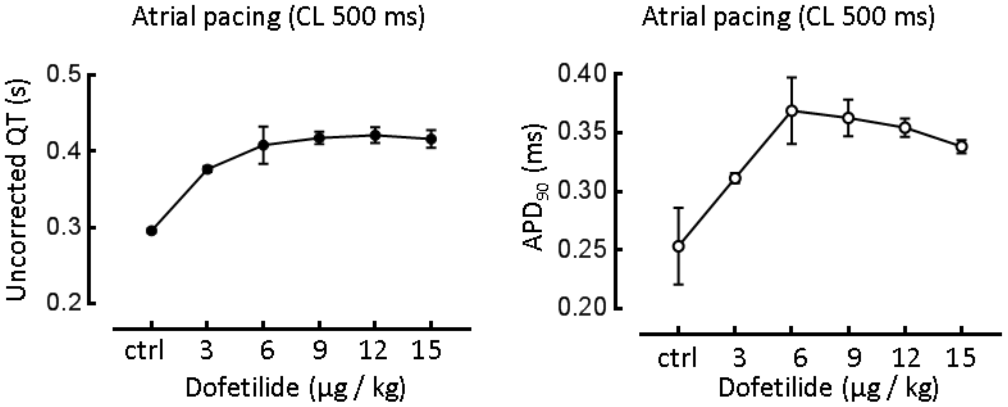

Supplemental Figure VIII. Dofetilide effects on QT interval and APD are independent of its effect on heart rate. Effect of dofetilide on QT interval (left) and action potential duration (right) during atrial pacing. Paired data from *N* = 3 animals. For QT interval, $p = 0.036$. For APD_{90}, $p = 0.014$. For both comparisons, Friedman test.

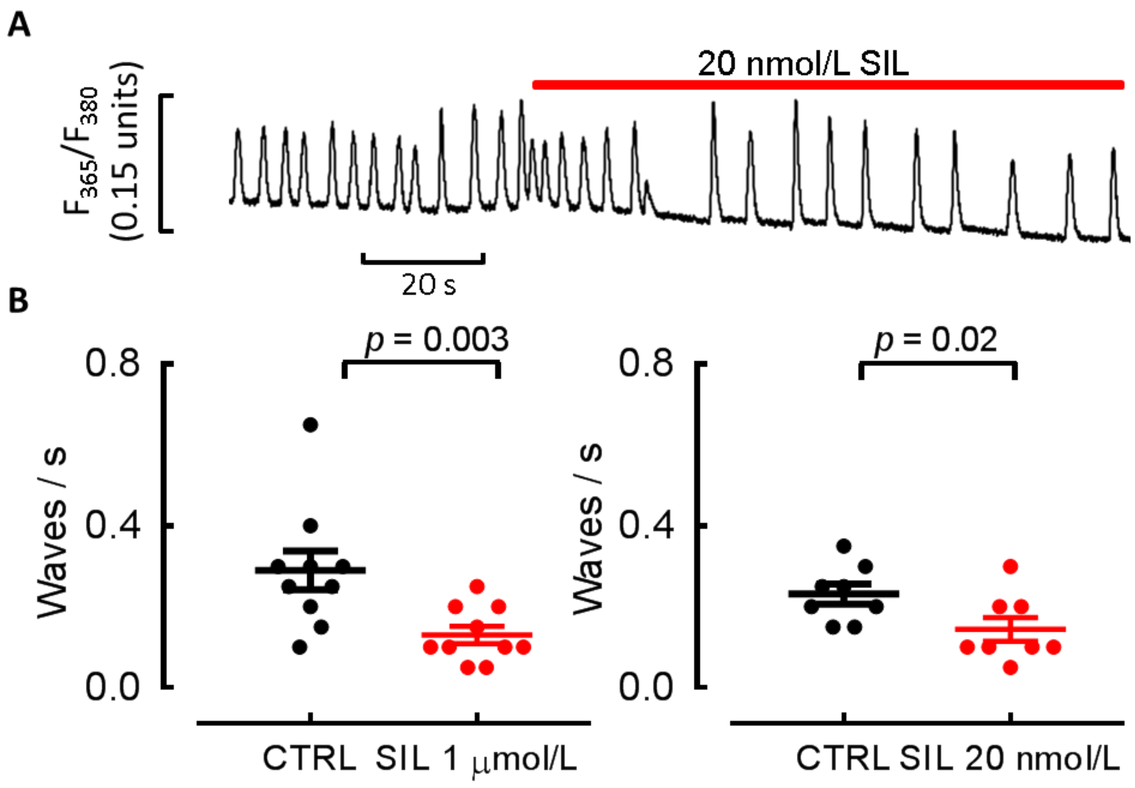

Supplemental Fig IX. Effects of different sildenafil concentrations on spontaneous Ca waves in unpatched cells. A. Representative Ca^{2+} recording of an unpatched cell exposed to high Ca^{2+} (15 mmol/L). Sildenafil is applied at lower concentration (20 nmol/L). B. Mean wave frequency in unpatched cells in high Ca^{2+}. Two concentrations of sildenafil are tested (left, 1 μmol/L, and 20 nmol/L, right). For 1 μmol/L sildenafil; paired data from 10 cells / 5 animals. For 20 nmol/L sildenafil; paired data from 8 cells / 2 animals. Paired t test for all comparisons.

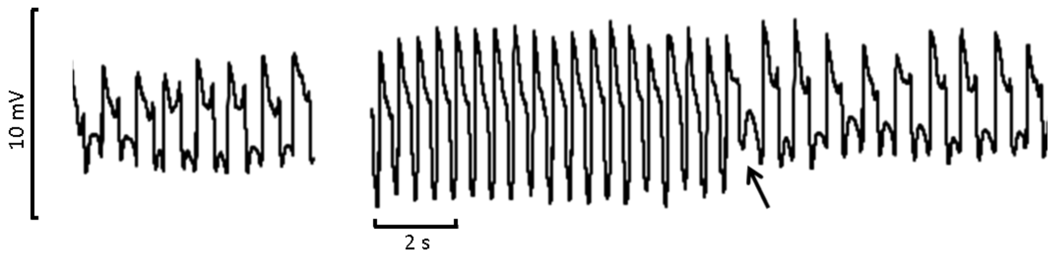

Supplemental Fig X. Left ventricular endocardial monophasic action potential recordings in a sheep treated with dofetilide (15 µg/kg). DADs are observed during sinus rhythm (left), and their amplitude is increased immediately after rapid pacing (arrow, right).

Supplemental tables

Parameter	Control	Dofetilide (max. dose)	*p* value	*N* (animals)
QT interval (ms)	409 ± 10	662 ±26	< 0.00001	11
T_{PEAK} to T_{END} interval (ms)	36.2 ± 3.2	111.5 ± 19.7	< 0.01	11
Heart rate (bpm)	84.1 ± 4.1	74.6 ± 2.7	< 0.01	11
Action potential duration (ms)	264 ±16	445 ± 29	< 0.0001	6
Systolic BP (mmHg)	89 ± 6	81 ± 5	< 0.01	11
Diastolic BP (mmHg)	50 ± 5	46 ± 5	0.11	11

Supplemental table I. Effect of dofetilide on *in vivo* parameters**.** Values are given for the maximum dose of dofetilide used in each animal (until TdP is observed, up to maximum 15 μg/kg). Paired t test for all comparisons.

Parameter	Control	Sildenafil	*p* value	*N* (animals)
QT interval (ms)	610 ± 34	581 ± 30	0.170	8
Heart rate (bpm)	74.9 ± 3.3	69.3 ± 3.5	0.007	8
PR interval (ms)	100 ± 5	103 ± 4	0.173	7
QRS duration (ms)	46 ± 1	44 ± 2	0.11	7
T_{PEAK} to T_{END} interval (ms)	109 ± 21	97 ± 19	0.174	7
Action potential duration (ms)	431 ± 43	413 ± 40	0.886	4
Systolic BP (mmHg)	83 ± 7	62 ± 3	0.008	7
Diastolic BP (mmHg)	48 ± 7	28 ± 2	0.0251	7

Supplemental table II. Effect of sildenafil on *in vivo* ECG parameters, monophasic action potential, heart rate and blood pressure. Control values (in dofetilide) were obtained immediately before administration of sildenafil. For action potential duration, Wilcoxon matched pairs test. For all other comparisons, paired t test.

**Parameter**	**1.8 mmol/L Ca^{2+}**	**10 – 15 mmol/L Ca^{2+}**	***p* value**	***N* (cells)**	***N* (animals)**
Normalized Ca^{2+} transient amplitude	1	1.96 ± 0.29	<0.005	54	28
k_{CAFF} (s^{-1})	0.51 ± 0.06	0.50 ± 0.09	1.00	14-17	8-12
k_{SYS} (s^{-1})	3.46 ± 0.27	6.02 ± 0.45	<0.0001	36	27
k_{SERCA} (s^{-1})	2.48 ± 0.36	5.94 ± 0.76	<0.0005	10-14	6-10
SR content (µmol/L)	44.3 ± 12.6	104.4 ± 14.6	<0.005	18-19	12-16

Supplemental table III. Effects of raising external Ca^{2+} on cellular Ca^{2+} handling. Paired t test for Ca^{2+} transient amplitude and k_{SYS} (s^{-1}). Unpaired t test for k_{CAFF} (s^{-1}), k_{SERCA} (s^{-1}) and SR content (µmol/L).

Parameter	Control	KT5823	*p* value	*n* (cells)	N (animals)
Peak $I_{Ca\text{-}L}$ (pA/pF)	3.84 ± 0.33	3.41 ± 0.33	0.54	19 - 83	6 - 44
Integrated $I_{Ca\text{-}L}$ (µmol/L)	2.00 ± 0.15	1.65±0.15	0.28	19 - 84	6 - 37
Ca^{2+} transient amplitude (F_{365}/F_{380})	0.27 ± 0.06	0.23 ± 0.07	0.73	11 - 13	4 - 5
k_{SYS} (s^{-1})	3.54 ± 0.65	3.80 ± 0.56	0.77	9 - 11	4 - 4

Supplemental table IV. Effects of KT5823 on cellular Ca^{2+} handling (1.8 mmol/L Ca^{2+}). Unpaired t test for all comparisons.

Parameter	KT5823	KT5823 + Sildenafil	*p* value	*n* (cells)	N (animals)
Normalized peak $I_{Ca\text{-}L}$	1	1.10 ± 0.38	0.80	11	6
Normalized integrated $I_{Ca\text{-}L}$	1	0.93 ± 0.16	0.66	11	6
Normalized Ca^{2+} transient amplitude	1	1.28 ± 0.30	0.39	6	5

Supplemental table V. Effects of sildenafil on cellular Ca^{2+} handling in the presence of KT5823. Experiments were performed in 15 mmol/L Ca^{2+}. Paired t test for all comparisons.
