# Supplementary figures and images for: PDE5 Inhibition Suppresses Ventricular Arrhythmias by Reducing SR Ca2+ Content
Source: Circ Res. 2021 Jul 12;129(6):650–65. doi: 10.1161/CIRCRESAHA.121.318473 (PMC8409902; doi:10.1161/CIRCRESAHA.121.318473)

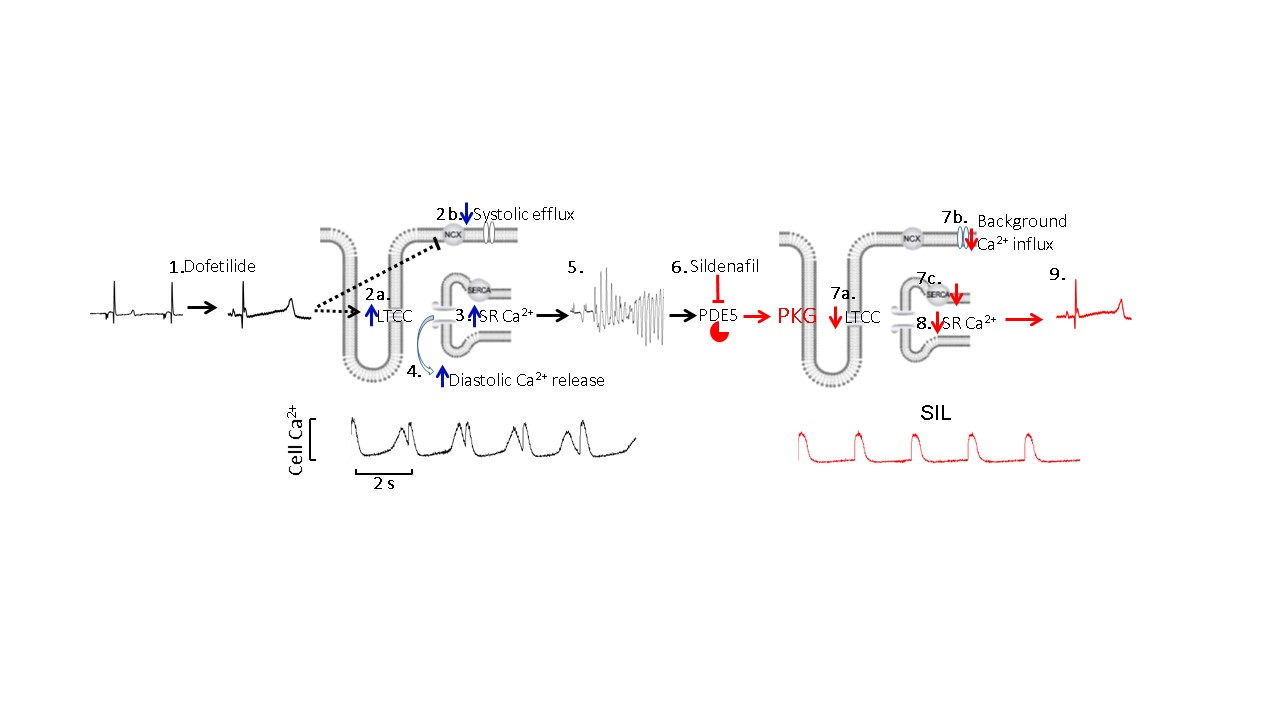

Supplement: Supplementary file 2 [file res-129-650-s002.jpg]
